# The near-field tsunami generated by the 15 January 2022 eruption of the Hunga Tonga-Hunga Ha’apai volcano and its impact on Tongatapu, Tonga

**DOI:** 10.1038/s41598-022-19486-w

**Published:** 2022-09-07

**Authors:** Kwanchai Pakoksung, Anawat Suppasri, Fumihiko Imamura

**Affiliations:** International Research Institute of Disaster Science, Tohoku University, Sendai, 980-0845 Japan

**Keywords:** Volcanology, Natural hazards, Nonlinear phenomena, Computational science, Applied mathematics

## Abstract

On 15 January 2022 at 04:15 UTC, the Hunga Tonga-Hunga Ha’apai (HTHH) volcano in Tonga produced a massive eruption that triggered a transoceanic tsunami generated by the coupled ocean and atmospheric shock wave produced during the explosion. The tsunami first reached the coast of Tonga and eventually reached many coasts around the world. This volcano previously underwent a massive eruption in 1100 AD, and an eruption occurs approximately every 1000 years. The 2022 HTHH event provides an opportunity to study a major volcanically generated tsunami that caused substantial damage. In this study, we present a numerical simulation of a tsunami with a state-of-the-art numerical model based on a submarine explosion scenario. We constrain the geometry and magnitude of the explosion energy source based on analyses of pre- and post-event satellite images, which demonstrate that the explosion magnitude varied from 1 to 90 megatons of trinitrotoluene (Mt). Estimated submarine explosion geometries result in a suitable explosion magnitude of approximately 25 Mt, as determined with the waveform from the tide gauge in the time and frequency domains. The tsunami wave first reached the northwestern part of Tonga’s Tongatapu within 10 min, with a maximum runup height of approximately 15 m, and covered the whole of Tongatapu within 30 min. Finally, the numerical simulation provides deep insights into the physical volcanic explosion processes and improves our understanding and forecasting capabilities of frequent and catastrophic tsunamis caused by submarine volcanic explosions.

## Introduction

**Figure 1:**
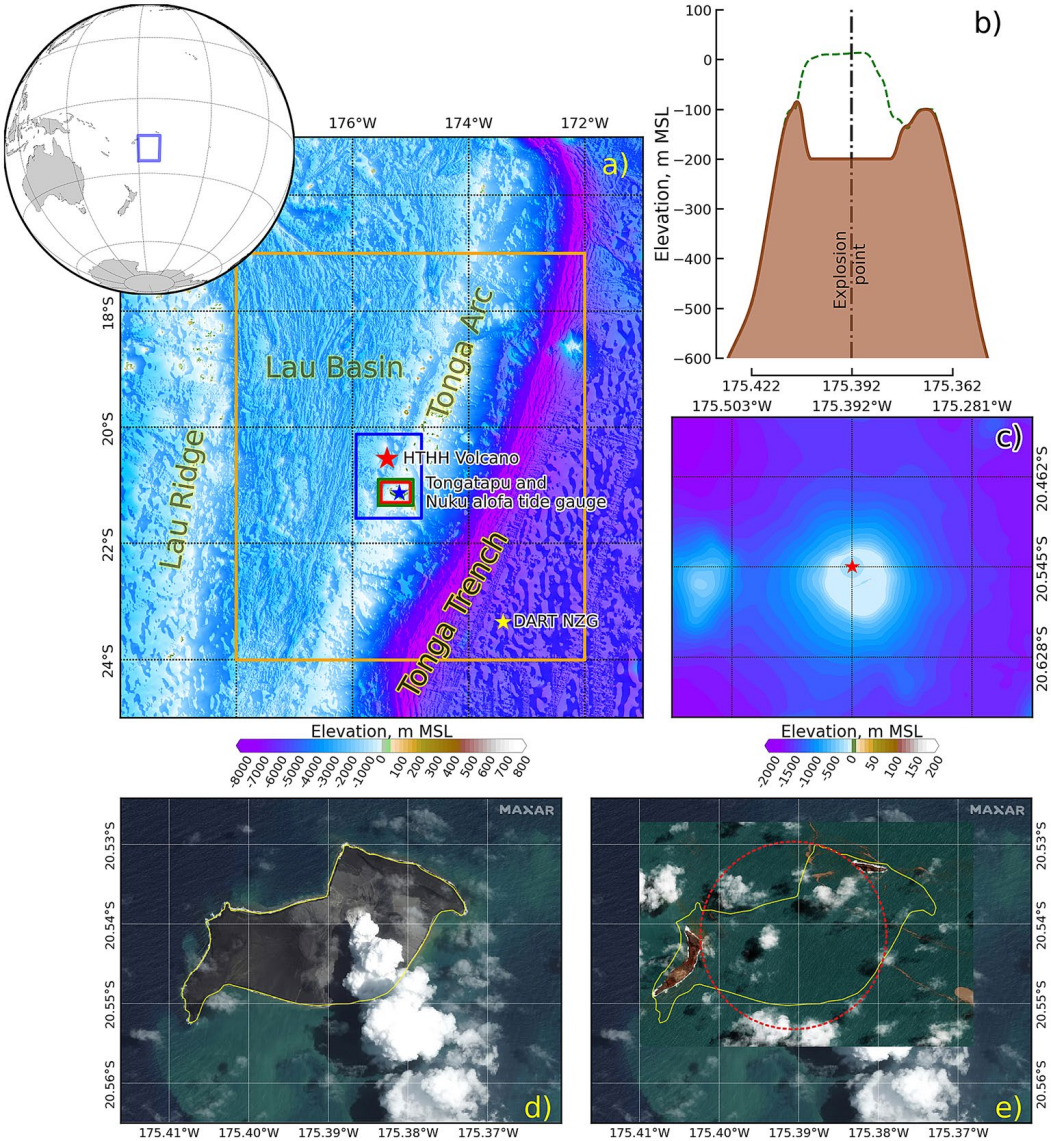
Study area. (**a**) Location of the Hunga Tonga-Hunga Ha’apai (HTHH) volcano (red star); Tonga’s Tongatapu and the selected observation point on the tide gauge (Nuku’alofa) are represented by the blue star, the yellow star is the selected observation point on the New Zealand DART station (NZG), and the 4 colored boxes (orange, blue, green, and red) are the areas used to simulate the tsunami, based on 60, 15, 3, and 1 arcsecond, respectively. (**b**) The profile of HTHH volcano (at 20.545$^{\circ }$S) before (green dashed line) and after (brown line) the eruption. (**c**) Topographic data (1 arcsecond) around HTHH volcano showing that the subduction area around the center of the eruption (red star) was hypothesized by the satellite image between (**d**,**e**). (**d**) Satellite image of the HTHH volcano before the eruption on 6 January 2022 by MAXAR, where the yellow line is the shoreline of the volcanic island. (**e**) Satellite image of the HTHH volcano after the eruption on 18 January 2022, for which the red dashed cycle was hypothesized to be the subduction area of this event. The satellite image from 2022 MAXAR Technologies were downloaded from ALASKA PUBLIC MEDIA^5^ website (https://www.alaskapublic.org/2022/01/18/nasa-scientists-estimate-tonga-blast-at-10-megatons). The figure was generated by using a QGIS software^11^, version 3.16.15-Hannover (http://www.qgis.org).

Tsunamis generated by submarine volcanic eruption explosions compose only a small percentage of all recorded tsunamis. A tsunami can be generated by the eruption of a submarine volcano, which disturbs the sea surface. The resulting tsunamis are of particular interest for evaluation and forecasting research. On 15 January 2022 at 04:15 UTC, the Hunga Tonga-Hunga Ha’apai (HTHH) volcano erupted^1^; the volcano had previously experienced a massive eruption in 1100 AD, and a massive eruption has occurred approximately every 1000 years^2,3^. The HTHH volcano is in the Tonga Islands in the southwestern Pacific Ocean, as shown in Fig. 1a. The eruption explosion power was reported as the energy released by a magnitude 5.8 earthquake^1^ and was equivalent to 4-18 megatons of trinitrotoluene (Mt)^4^. Additionally, a report from NPR^5^ mentioned that the energy of this eruption was approximately 10 Mt. Furthermore, a range of 9-37 Mt was estimated for this event based on the analysis of ionospheric observations that suggested that the main explosion, among 5 explosions during the eruption, occurred at 04:16:20 UTC^6^. The tsunami triggered by the eruption of this submarine volcano reached a height of 83 cm in the Nuku’alofa area (see Fig. 1a) on Tonga’s Tongatapu^7^. The Tongan government reported that the maximum water level in the northwestern area of Tongatapu was approximately 15 m^3^. In the case of the 2022 HTHH eruption explosion, the source of the tsunami was likely very large Lamb (pressure pulse) atmospheric waves^8–10^. A major challenge is the development of a numerical model to predict the impacts of tsunamis.

Tsunamis generated by submarine volcanic explosions, as in the case of the 2022 HTHH eruption, are controlled by several physical parameters, such as water depth, eruption vent size, explosion energy, and magma interaction, which are used to define the explosion itself^12–21^. The explosion process in the initial crater results in the formation of a similar cavity at the water surface with a cylindrical bore. Next, the cylindrical bore expands radially to form a leading wave, followed by a wave trough. The initial water surface displacement corresponds to the maximum height of the bore that can be empirically estimated by a function of explosion energy^12,21–24^. The initial downward water displacement follows the upward displacement and forms a steep cone in the center of the bore^18^. The cone collapses and generates a second bore, initiating tsunami propagation in the sea, as presented in the numerical model in a previous study^21,25,26^.

The water surface displacement model generated by a submarine explosion would be difficult to incorporate into initial conditions for a tsunami simulation. The suggested method is usually performed with an empirical model to estimate the change in the water surface at some distance from the eruption point, as mentioned by Le Mehaute^12^ and Le Mehaute and Wang^17^, and includes the initial water displacement. The model for initial water displacement is a function of the maximum water level, the radius of the explosion, and the radial distance from the source point. This model is suitable for submarine explosions^15,27^. This suggested modeling approach was used to generate the initial conditions of the tsunami model based on the nonlinear wave model in the case of the 1996 eruption of the Karymsky volcano^27^.

In the present study, we develop a model for the tsunami caused by the explosion involved in the main eruption of the 2022 HTHH volcano. By varying the explosion energy, the model specifically simulates the explosion source of the submarine volcano. This tsunami may have been generated by other sources, such as prearrival tsunamis observed in Japan and other countries caused by the air-water interaction or by submarine landslides. However, this simulation is still applicable in this study (which considers only the main explosion) when verifying the simulation result with only near-field data. Then, we use the empirical model for the initial surface displacement in this study to fill the knowledge gap in the previous study and implement the modified empirical model for the real event, namely, the 2022 HTHH volcanic eruption. The modified empirical model was limited to only a function of explosion energy and water depth. Subsequently, the modified empirical model is used to investigate the suitable tsunami source for this event. The initial water level generated by the modified empirical model is input into the tsunami propagation model (TUNAMI model with Boussinesq-type equations) that was transformed into the geographical (spherical) coordinate system in this study, and the tsunami hazard characteristics, maximum water level, and arrival time around Tongatapu, Tonga, are obtained. The TUNAMI model has been extensively benchmarked and used in several tsunami case studies^24,28–30^. The accuracy of the model is validated by using the observed waveform at the tide gauge because of the limited availability of field observation data. Detailed information about tsunami hazards provides scientific guidance for designing and implementing tsunami prevention structures and developing future tsunami evacuation plans.

## Results

### Initial water level, tsunami wave generation, and tsunami wave propagation

**Figure 2:**
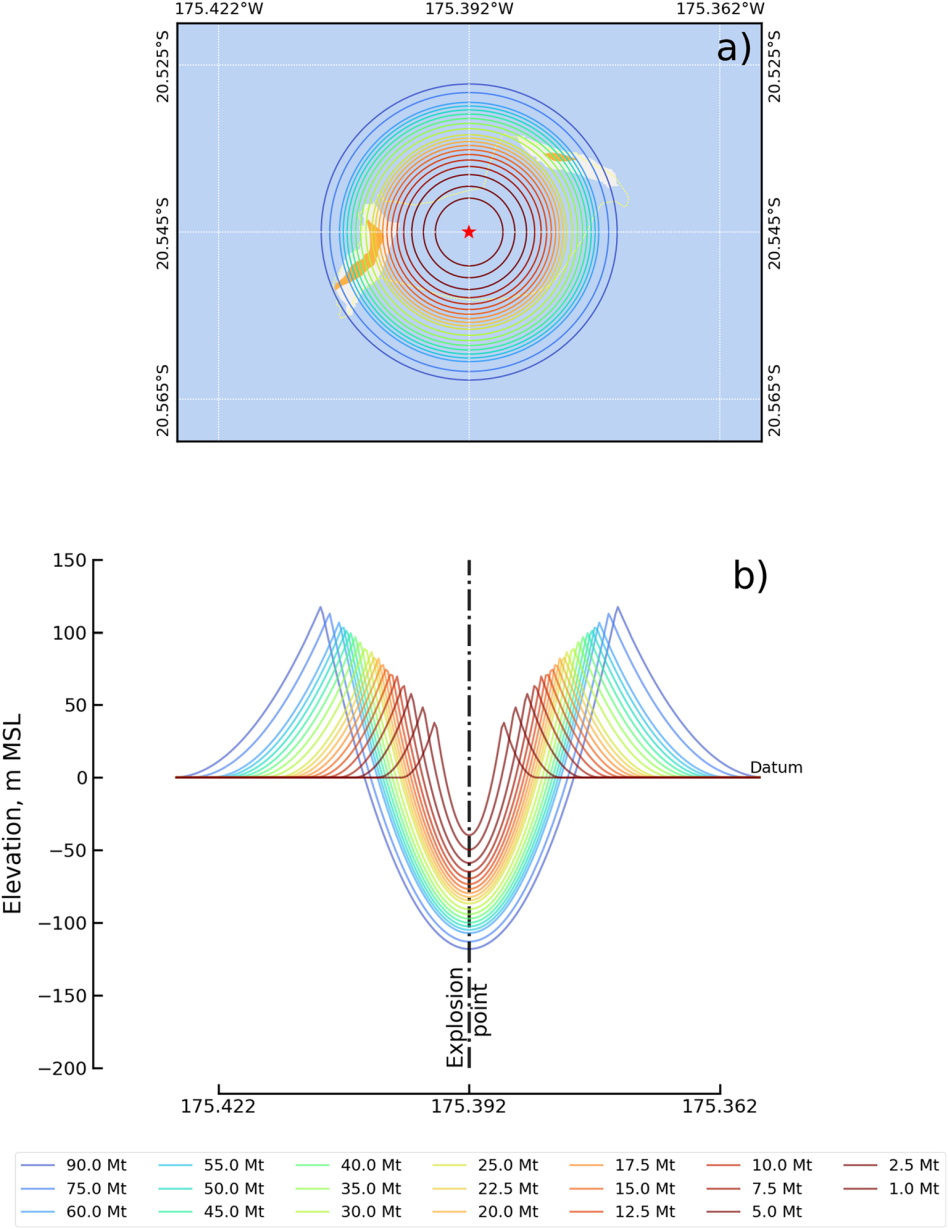
Estimated initial water level based on the explosion energy that varied from 1 to 90 megatons of trinitrotoluene (Mt) with the deep sea depth condition. (**a**) Size of the water cavity (highest point) produced from the explosion energy. (**b**) Profile of the initial water level at 20.545$^{\circ }$S.

The predicted results from 20 scenarios of the 2022 HTHH volcanic eruption explosion and the tsunami generated by the proposed formula are presented in this section. The 20 explosion scenarios, with explosion magnitudes varying from 1 to 90 Mt, are presented in Fig. 2. Based on Eq. 1, the explosion diameter, which corresponded to the explosion energy, varied from 875 to 3,920 m, as shown in Fig. 2a. Figure 2b presents the maximum initial water level based on Eq. 2 with respect to the deep sea depth condition and the parabolic shape distribution of the initial water level in the profile at the assumed explosion point of the volcano (175.392$^{\circ }$W, 20.454$^{\circ }$S) based on Eq. 3, which is the original contribution of this study. The calculated initial water levels varied from 40 to 120 m for the lowest and highest explosion energies of the candidate scenarios, respectively.

**Figure 3:**
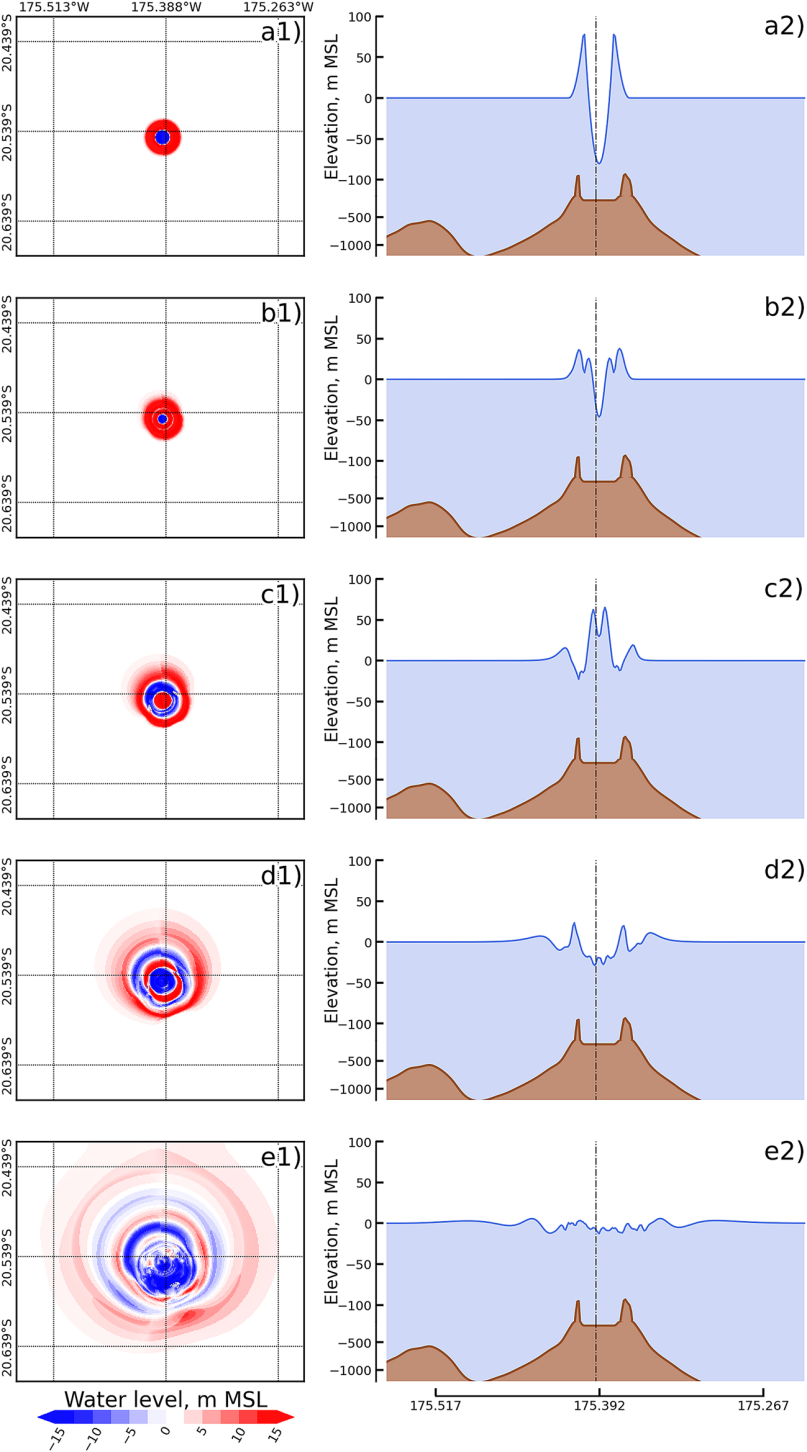
Tsunami generation for the scenario of 25 Mt with the deep sea depth condition: the first column presents the spatial data, and second column presents the profile at 20.545$^{\circ }$S: (**a**) 0 second, (**b**) 10 second, (**c**) 30 second, (**d**) 1 min, and (**e**) 2 min.

Tsunami generation was based on the size of the explosion, maximum initial water level, and parabolic distribution, determined from Eqs. 1, 2, and 3. Figure 3 presents the tsunami generation in map view (first column) and profile (second column) for the example of the 25 Mt candidate scenario with an explosion size of 2,558.744 m and a maximum initial water level of 86.895 m (based on the deep sea depth condition); the propagation is shown at different times, namely, 0, 10, 30, 60, and 120 seconds, after the assumed main explosion at 4:16:30 UTC. The tsunami initially formed a symmetrical parabolic shape (see Fig. 3a), after which the second wave was generated and moved from the center of the explosion point (see Fig. 3b). After the second wave was generated, due to the continuity equation (Eq. 4) and momentum equation (Eqs. 5 and 6), the tsunami propagated forward to the northwestern and southeastern sides faster than to the northeastern and southwestern sides. On both the northeastern and southwestern sides, the tsunami moved more slowly due to Hunga Tonga Island and Hunga Ha’apai Island because both islands are the old calderas of this volcano^31,32^, as shown in Fig. 3c,d. After 120 seconds, as shown in Fig. 3e, the third wave was generated, and the first wave propagated to the deep area. The wave formed a similar symmetrical shape in the northern area of the explosion point due to the presence of a deep area caused by the refraction impact from the change in bathymetry^33,34^.In deep sea areas, tsunami waves form symmetric shapes based on increasing water depth, which impacts the momentum equation (Eqs. 5 and 6). The 2nd, 3rd, and 5th terms of the momentum equation in this study have a few effects in deep water; therefore, the wave in the deep area can propagate on the sea by using the hydrostatic force (4th and 7th term of Eqs.  5 and 6). On the southern side of the volcano, the first tsunami wave was diffracted by the seamounts to form an asymmetrical shape.

**Figure 4:**
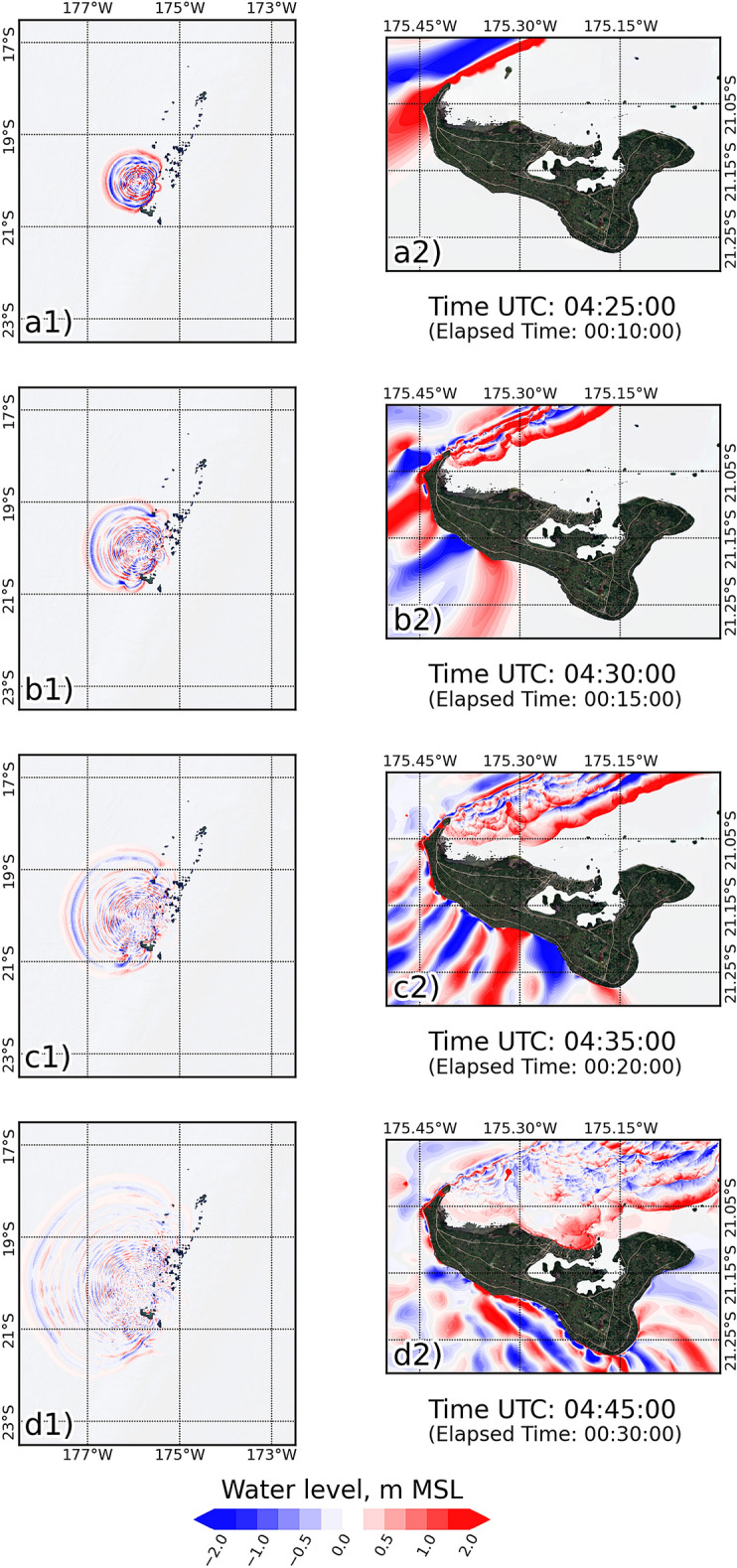
Tsunami propagation for the scenario of 25 Mt with the deep sea depth condition, showing the largest region in the 1st column and the smallest region in 2nd column: (**a**) 10 min, (**b**) 20 min, (**c**) 25 min, and (**d**) 30 min. The maps were created with a QGIS software^11^, version 3.16.15-Hannover (http://www.qgis.org), and the satellite image for basemap was downloaded from QuickMapServices plugin (https://github.com/nextgis/quickmapservices) through the QGIS^11^.

Tsunami propagation is based on the initial water level distribution, which comes from the proposed formula in this study and is driven by the TUNAMI model that was transformed into the geographical coordinate system. Figure 4 shows the spatial tsunami propagation in two regions; the first column shows the largest region (60 arcseconds resolution), and the second column shows the smallest region (1 arcsecond resolution). In the example, tsunami propagation at the different elapsed times after the assumed main explosion (4:16:30 UTC), namely, 10, 15, 20, and 30 min, is shown in Fig. 4 for the 25 Mt candidate scenario (based on the deep sea depth condition). The tsunami propagation in the largest region of the simulation shows that the tsunami wave on the west side of the volcano propagated faster than the wave on the east side (see Fig. 4a1,b1). The faster tsunami wave on the west side was caused by the deeper bathymetry in the west due to the volcano located in the Tonga Arc, as shown in Fig. 1a. West of the volcano (or simulation area) is the Lau Basin, which is located between the Lau Ridge and Tonga Arc^35,36^, where the tsunami wave can propagate more easily due to the momentum equation, as mentioned above. In contrast, on the east side of the volcano (or simulation area), tsunami waves propagate slowly due to the shallow area, which is a location of seamounts and islands along the Tonga Arc. After the first wave propagated in the Lau Basin, the reflected wave occurred in the seamounts and island group along the Tonga Arc and propagated westward, as shown in Fig. 4c1–d1.

In the smallest simulation area, the tsunami wave arrived first at the northwestern side of Tongatapu (see Fig. 4a2). Next, the tsunami wave was diffracted by the island, and the diffraction wave propagated northward and southward. The diffracted wave in the southern area was faster than the wave in the north, as presented in Fig. 4b2–d2. The northwestern area of Tongatapu shows a small inundation area after the elapsed time of 15 min due to the first wave. The difference in the propagation velocity of the tsunami wave between the northern and southern areas was caused by the topography of the island, which affected the wave movement in Eqs. 5 and 6 in the tsunami simulation. The northern area is the location of seamounts and coral reefs, which cause the sea in this area to be shallow^37^. Therefore, the tsunami wave propagating in this area was slow, and the waveform developed a sharp front^38^. On the other hand, the southward wave on the island propagated quickly, and the waveform had a smooth front due to the deeper bathymetry on this side compared to that on the north side.

### Waveform analysis results

**Figure 5:**
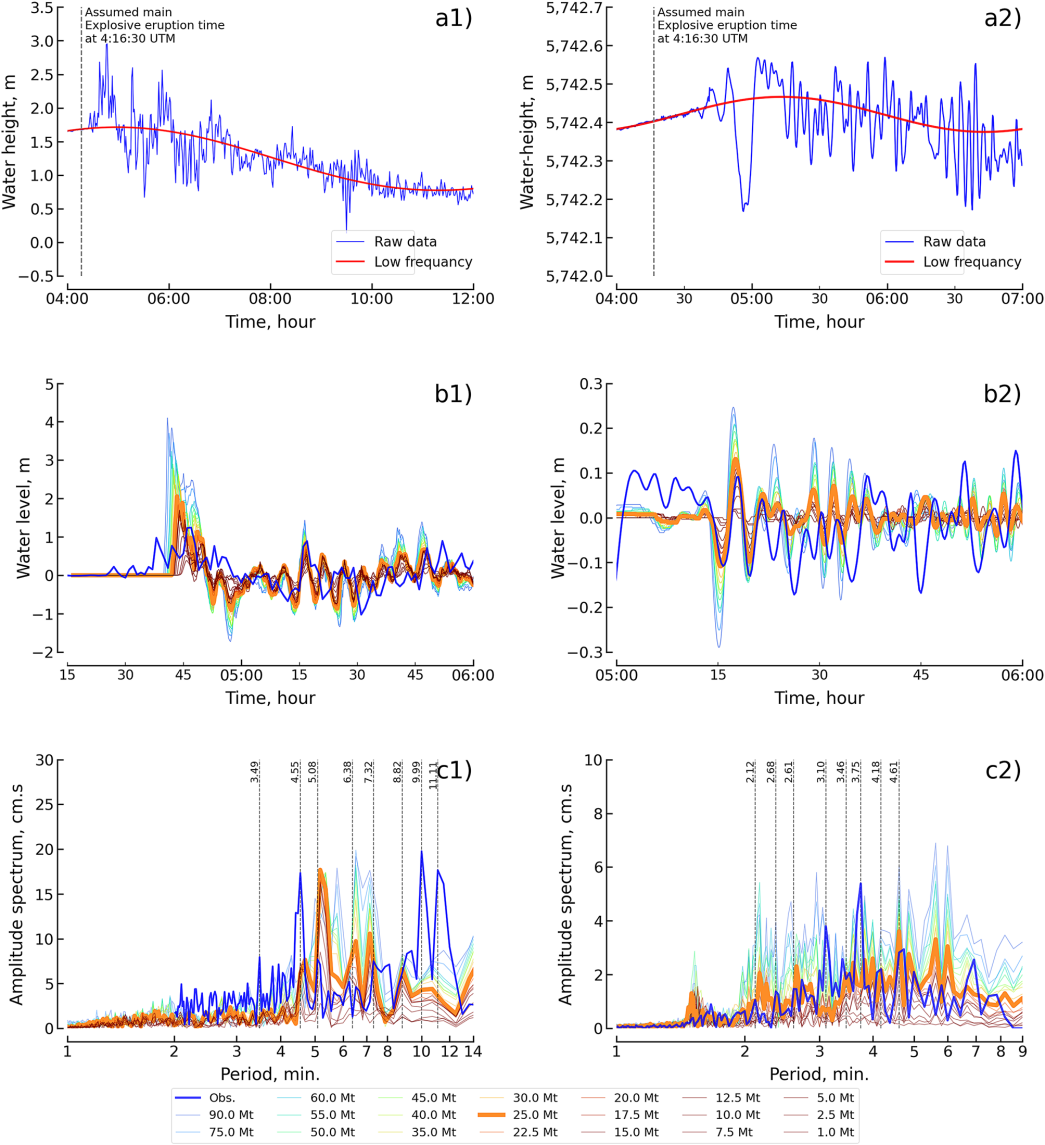
Waveform and wave characteristics of observation and simulation results, showing the Nuku’alofa station in the 1st column and the NZG station in the 2nd column. (**a**) Waveform beginning at 4:00 UTC on 15 January 2022. (**b**) Filtered time series data from the high-pass filter compared to the simulated waveforms of the 20 explosion scenarios with the deep sea depth condition. (**c**) Comparison of wave characteristics in the amplitude spectrum.

The tsunami measured height at the tide gauge is compared with simulated data to validate the numerical simulation results. Two tide gauges are close to the HTHH volcano: the Nuku’alofa and NZG gauges, as shown in Fig. 1a. Nuku’alofa is located in the shallow water area, while the NZG gauge is located in the deep sea area. The Tonga meteorological station installed the Nuku’alofa tide gauge near the north shore of Nuku’alofa, the capital city of Tonga. This tide gauge recorded the sea water surface elevation during the 2022 HTHH tsunami at a sampling rate of 1 min, as shown in Fig. 5a1, and the data were provided by VLIZ^39^ and Greenwood^40^. We selected a gauge in the deep sea to validate the simulation results because the deep area was not affected by the friction force of a shallow reef mount. The GeoNet program of New Zealand’s Institute of Geological and Nuclear Science (GNS) installed the NZG tide gauge in the deep sea that completely recorded this event^41^. The tide gauge recorded the sea water surface elevation during the 2022 HTHH tsunami at a sampling rate of 15 seconds, as shown in Fig. 5a2, and the NZG station is the closest deep sea station to the source. After quality control of the raw data, we applied a high-pass filter with a cutoff of 15 min (Nuku’alofa) and 8 min (NZG) to remove the tidal signals^42,43^, and the low-frequency trend is shown in Fig. 5a. The filtered data are the residuals from the difference between the raw data and the low-frequency data. The detided data at the tidal gauge were compared with the simulated waveform at the corresponding location.

We show our results from the tsunami simulation based on the proposed method to estimate the initial condition of the water level generated by the 2022 HTHH eruption explosion. The simulated tsunami waveforms from 20 explosion magnitude (Mt) scenarios with the deep sea depth condition at the Nuku’alofa and NZG tide gauge were compared with measured waveforms, as shown in Fig. 5b. In the time domain, the 25 Mt simulated waveform generally shows reasonable agreement with the waveforms measured on both tide gauges. The reasonable volcanic explosion magnitude of 25 Mt agrees with the previous report by Astafyeva et al.^6^. At the Nuku’alofa tide gauge, the first peak amplitude is overestimated by approximately 1 m and the water level is in a faster phase than the measured waveform; however, the following pattern of the simulated waveforms is quite consistent with the pattern of the measured waveform. In contrast, the arrival time of the simulated waveform is delayed after the measured waveform by approximately 15 min. At the NZG tide gauge, the peak of the reasonable fit (from 05:15 to 05:22) is almost of the same amplitude as the water level in the small, faster-moving phase versus the measured waveform; however, the subsequent pattern of the simulated waveforms is quite poorly predicted with respect to pattern of the measured waveform. The poorly prediction of the subsequent waveform after the first and second peaks might be caused by the subsequent explosion, as mentioned by Astafyeva et al.^6^. In contrast, the arrival time of the simulated waveform is delayed after the measured waveform by approximately 35 min. The discrepancies between the simulated and measured tsunami waveforms mainly result from bathymetric and topographic grids. We lack high-resolution bathymetry and topography data around the tide gauge. However, the simulated results still have satisfactory performance. Additionally, the discrepancies (e.g., early arrival wave) are from other tsunami sources that were not considered in this study.

In the spectral domain, the amplitude spectra of the simulated waveforms for 20 scenarios of the explosion magnitude from the 2022 HTHH volcano were compared with the amplitude spectra of the measured waveform, as shown in Fig. 5c. The amplitude spectrum of the simulated waveform of 25 Mt matches well with the amplitude spectrum of the measured waveform which agrees fairly well with the spectrum for comparison in the time domain. The peak spectrum at the Naku’alofa tide gauge was considered for the periods of approximately 3.49, 4.55, 5.08, 7.32, 9.99, and 11.11 min. The explosion scenario (25 Mt) correlates well with the reproduced peak close to the measured amplitude spectra with a different period of approximately of 30 seconds. The peak spectrum at the NZG gauge was considered for the periods of approximately 2.12, 2.68, 2.61, 3.10, 3.75, 4.18, and 4.61 min. The explosion scenario (25 Mt) correlates well with the reproduced peak close to the amplitude spectra recorded at only the peak periods of 2.12, 2.61, 4.18, and 4.61 min. The difference between most peak periods of simulated and measured signals is more than 10%, which does not agree well with the criterion values mentioned by Cortes et al.^44^, Wang et al.^45^, and Ren et al.^43^. The discrepancies in the spectra mainly result from the complex mechanism involved at the source of this event, which might have included a magma interation, submarine landslide, pyroclastic flow, debris flow, caldera collapse, or atmospheric wave pressure^46^, while this study considered only the submarine explosion as the source.

### Tsunami hazard

**Figure 6:**
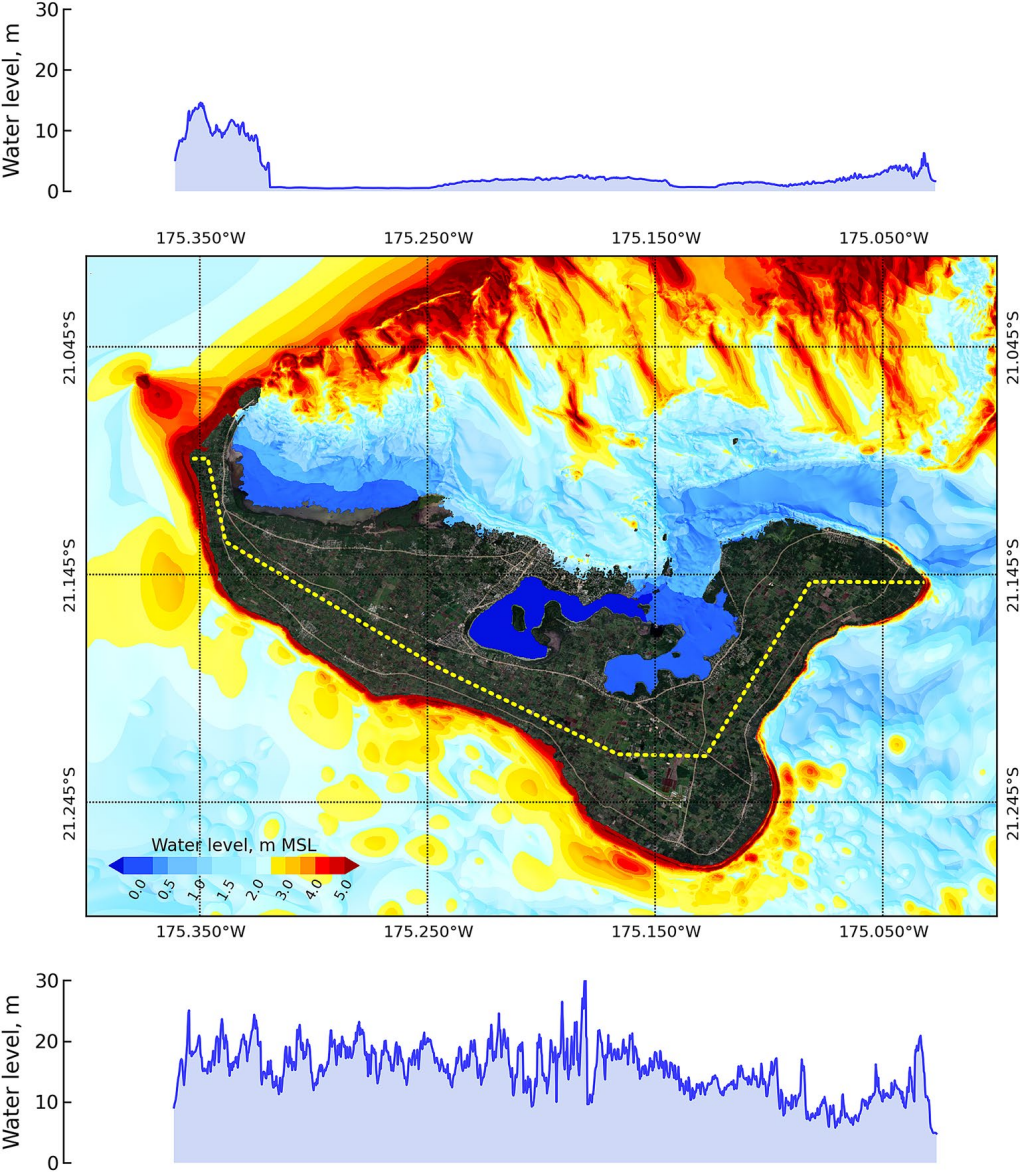
Maximum water level around Tongatapu after 3 hours of simulation for the 25 Mt explosion source at the HTHH volcano on the deep sea depth condition. The middle panel shows the spatial distribution of the maximum water level. The top panel shows the maximum water level at the coastline to the north of the island, and the lower panel shows the maximum water level to the south. The map was created with a QGIS software^11^, version 3.16.15-Hannover (http://www.qgis.org), and the satellite image for basemap was downloaded from QuickMapServices plugin (https://github.com/nextgis/quickmapservices) through the QGIS^11^.

Figure 6 presents the maximum water level for the smallest region (around Tongatapu) after 3 hours in the tsunami model for the 25 Mt scenario with respect to the deep sea depth condition at the source of the explosion at HTHH in 2022. The middle panel of the figure shows the spatial distribution of the maximum water level, while the top and bottom panels show the profile of the maximum water level along the coastline of the island in the north and south, respectively. The north side and the south side are divided by the yellow dashed line, as shown in the middle panel of this figure. Spatially, the north side of the island in the sea showed an average water level height of approximately 10 m in the large area that is far from the north shore, approximately 6 km, while the area close to the north shore had an average water level of approximately 1.5 m. The difference in the water level in the areas far from and close to the shore due to the bathymetry in the northern area defines the shallow depth zone. The shallow depth zone in the northern part of the island, which is located by a large area of coral reef^47^, acts as a natural barrier against tsunamis^48^. In the southern area, the water level height of approximately 15 m is along the area close to the shore, while the area far from the shore shows a water level that is less than 3.0 m. The profiling of the water level on the shore shows that the water level is lower in the northern area than in the south. On the north shore of the west side, the water level is locally reported to be 15 m^3^, which is close to the simulation result of the present study. Additionally, the simulation resulted in a small inundation area in the northwestern area.

**Figure 7:**
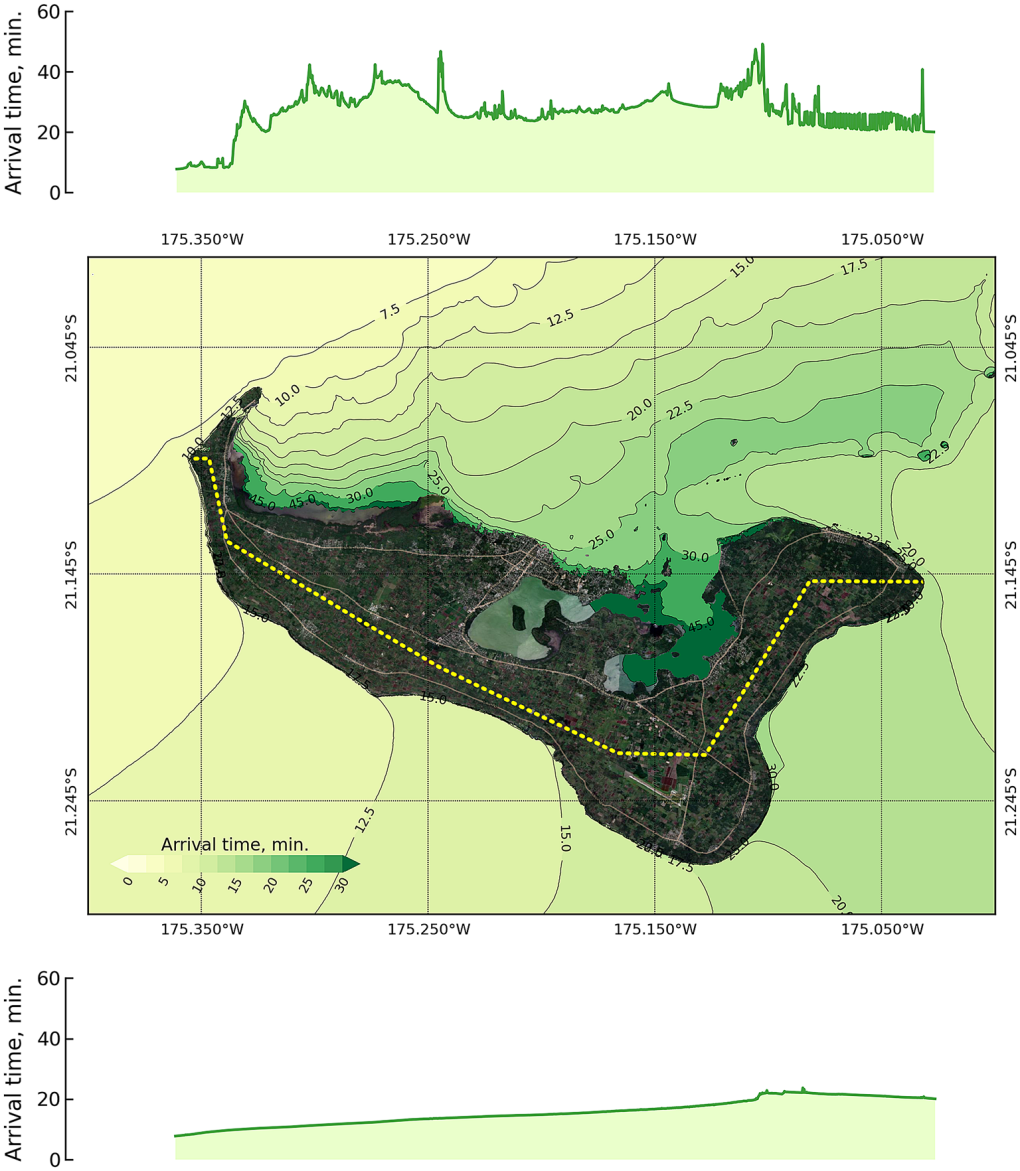
Arrival time of the tsunami around Tongatapu after 3 hours of simulation for the 25 Mt explosion source at the HTHH volcano on the deep sea depth condition. The middle panel shows the spatial distribution of the arrival time. The top panel shows the arrival time at the coastline to the north of the island, and the lower panel shows the arrival time to the south. The map was created with a QGIS software^11^, version 3.16.15-Hannover (http://www.qgis.org), and the satellite image for basemap was downloaded from QuickMapServices plugin (https://github.com/nextgis/quickmapservices) through the QGIS^11^.

Furthermore, a velocity decrease was observed over the main high-relief area, and the velocity variation produced an alteration in the concentric pattern of arrival times obtained from the 25 Mt candidate scenario covering the smallest domain area, as shown in Fig. 7. The distribution of arrival times appears marked by inflection lines in the propagation pattern, as shown in the middle panel of the figure. The first place struck by the tsunami wave was the northwestern area of the island. The large area of coral reef on Tongatapu shelf magnified the amplitude; however, these coral reefs were responsible for wave deceleration and therefore produced a delayed arrival along the coastline in the north shore area, as presented by the profiling in the top panel of this figure. In the southern area, the tsunami wave arrived in this area due to the wave diffraction from the northwestern side of the island along with the deep-sea conditions. The profile of the arrival time in the south is shown in the bottom panel of this figure. Therefore, the arrival time in the northern shore area was slower than the arrival time in the southern shore area due to the impact of the coral reef^49^.

## Conclusion and discussion

Tsunamis associated with large explosive eruptions in submarine settings are generated by the rapid displacement (upward or downward) of the sea surface, and the possible mechanisms include submarine explosions, entry into the sea of pyroclastic flows or debris flows, submarine landslides, and caldera collapse^46^.

In this study, we present the numerical simulation of the 2022 HTHH volcanic eruption explosion that generated a tsunami that impacted Tonga’s Tongatapu by considering only the source of the submarine explosion. The initial sea surface displacement related to the explosion energy of the volcano was estimated by our modified empirical model. This modified empirical model was used to solve the discontinuity of the initial sea surface displacement for tsunami propagation modeling. The proposed empirical model was limited to the basic parabolic shape and only represented a function of explosion energy and water depth. For the simulation modeling, we used the TUNAMI model to obtain the tsunami propagation results. We modified the governing equation of the TUNAMI model with a Boussinesq-type equation and transformed it from the Cartesian coordinate system to the geographical (spherical) coordinate system based on Baba et al.^50^, which can be applied to a large area and is the spherical system that is closest to the real surface of the Earth. The Boussinesq-type equation was used to solve the dispersive term in the tsunami model for wave propagation in deep water. The tsunami simulation was related to the main explosion eruption at 04:15 UTC that was reported by the USGS^1^ and Astayeva et al.^6^.

Twenty candidate scenarios with the deep sea depth condition (varying from 1 to 90 Mt) for the explosion magnitude of the 2022 HTHH volcano were modeled, and the 25 Mt scenario revealed a suitable simulated waveform slightly close to the measured waveform at the Nuku’alofa and NZG gauge in the time and frequency domains. The proposed magnitude of 25 Mt in this study for this event agrees with the explosion energy reported by Astafyeva et al.^6^ and Wall^51^. This magnitude may be an overestimation in some peak periods, which should be considered in the amplitude spectra. Additionally, the simulation runup results at the shoreline in the northwestern part of the study area are close to the locally reported data of approximately 15 m. The early arrival wave before or during 04:15 UTC is from other tsunami sources that were not considered in this study.

The empirical model of maximum initial water level estimation related to the explosion energy and water depth was provided by Le Mehaute^12^, as given in Eq. 2. The water depth effects in the maximum initial water level estimation were assessed by the constant with 2 conditions, deep sea and shallow sea depth. This revealed that the maximum initial water level estimated by the shallow sea depth condition was approximately twice the water level estimated by the deep sea depth condition. The suggested explosion magnitude of this event based on the shallow sea depth condition was approximately 2.5 Mt, which might be smaller than the real situation. For the deep sea depth condition, it suggests the explosion magnitude of approximately 25 Mt, within the range suggested by Astafyeva et al.^6^ and Wall^51^. The existing empirical model of the initial water level generated by submarine explosions reproduced a waveform that was approximately one-fifth the size that produced with the modified empirical model (Eq. 3) at the same magnitude of explosion. The suggested explosion magnitude of this event based on the existing empirical model was approximately 150 Mt, which might be larger than the real situation.

The main limitation of this study is associated with the modeling of the explosion itself: the model used to estimate the initial sea surface displacement produces a more powerful explosion based on a function of explosion energy, and water depth, as the most important parameters^23,52^. Other parameters, such as eruption vent size and magma interaction, should be considered in estimation of initial sea surface displacement. The leading wave (first and second) is predicted well at NZG, while the subsequent waveform is predicted poorly due to the fact that we considered only the main explosion (1 instance) which occurred at 4:16:30 UTC on 15 January 2022. Furthermore, 5 explosions occurred during this event based on the ionospheric observation suggested by Astafyeva et al., 2022. We considered the subsequent waveform to be predicted poorly due to the subsequent explosion. We suggest adding the subsequent explosion to enhance the accuracy of the subsequent waveform after the first and second peaks. The assumption for the magnitude might result in an overestimation or underestimation in some areas, while the morphology of the crater might be influenced by a combination of vertical and horizontal explosions^53,54^. In future research, other sources, such as pyroclastic flows, debris flows, submarine landslides, caldera collapses, or atmospheric wave pressures, should be considered in numerical simulations of tsunamis for these events^46,55^.

## Methods

This study was supported by three elements. First, hypothesized submarine volcanic explosion modeling was used to identify the initial water level as a tsunami source. Second, the numerical model was used to reproduce the tsunami propagation based on the tsunami source. Finally, the model results were evaluated by the observed waveform in the time series and frequency domains. The frequency domain analysis is presented based on wave spectra estimated by the Fourier transform.

### Tsunami generation

We adopted a semianalytical method in which the dynamics of the submarine explosion were determined by simply imposing the initial water deformation for wave propagation modeling. The characteristic radius of the initial water displacement (*R*) in meters has a relationship with the explosion energy (*E*) in Joules with the empirical formula provided by Le Mehaute and Wang^17^. We modified the formula to change the *E* in megatons of trinitrotoluene (Mt) as follows:

1 $$\begin{aligned} R=4.37539\times 10^2 \cdot E^{1/3} \end{aligned}$$

The estimation of the maximum initial water level $(\eta _{0}$) in meters, related to the *E* in Joules and water depth (*d*) in meters through the constant (*c*), was provided by Le Mehaute^12^. The *c* is a function of the explosion energy and water depth, and is assumed to have one of two values. The first value was identified with the deep sea depth condition, while the second value was identified by the shallow sea depth condition. For this study, we modified the formula, changing the *E* in megatons of trinitrotoluene (Mt) as follows:

2 $$\begin{aligned} \eta _0=c \cdot E^{0.24} {\left\{ \begin{array}{ll} c=80.26395 &{}, 81.4164< d/E^{1/3} \le 2,449.11 \\ c=163.3343 &{}, 0 < d/E^{1/3} \le 81.4164 \\ \end{array}\right. } \end{aligned}$$

The distribution of the initial water level displacement ($\eta$) from the center of the explosion is related to the character radius (*R*), the maximum initial water level ($\eta _{0}$), and the radial distance from the source point (*r*). The existing model for estimating the distribution of initial water displacement (provided by Le Mehaute^12^) has limitations with the discontinued water surface distribution at $r=R$. The discontinued water surface distribution might carry a risk of numerical instability caused by the sharp wavefront in the TUNAMI model. We proposed a modified formula to solve the limitation of the existing formula, which is the original contribution of this study.

3 $$\begin{aligned} \eta (r,0)= {\left\{ \begin{array}{ll} \eta _{0}[2(\dfrac{r}{R})^2-1] &{}, \quad r \le R \\ \eta _{0}[2-\dfrac{r}{R}]^2 &{},\quad R < r \le 2R\\ 0 &{}, \quad r > 2R \end{array}\right. } \end{aligned}$$

We selected 20 different sizes (varying from 1 to 90 Mt) of explosion energy at the eruption location (see Fig. 1a) corresponding to the location of the 2022 HTHH submarine explosion to understand the suitable explosion energy for this event. We considered only the main explosion that occurred at 4:15 on 15 January 2022.

### Tsunami propagation modeling

To obtain the tsunami propagation results for different explosion magnitudes, numerical tsunami modeling was run by the TUNAMI model, which was first developed at Tohoku University^56,57^. The TUNAMI model operates using the nonlinear theory of the shallow water equation that is solved by a leap-frog and upwind scheme based on a cartesian coordinate system. The cartesian coordinate system is limited by a small area, with a small change in the spherical surface of the Earth^58,59^. We therefore modified the existing governing equation of the TUNAMI model to a geographic (spherical) coordinate system based on Baba et al.^50^, as given in Eqs. 4, 5, and 6. We also added a dispersion term with the Boussinesq-type equation into the governing equation based on Baba et al.^50^ but with a slight modification. The finite difference method was applied to solve the modified governing equation.

4 $$\begin{aligned}&\dfrac{\partial \eta }{\partial t}+\dfrac{1}{R \, cos \, \theta } \left[ \dfrac{\partial M}{\partial \lambda }+\dfrac{N \, cos\, \theta }{\partial \theta }\right] =0 \end{aligned}$$

5 $$\begin{aligned}{}&\begin{aligned} \dfrac{\partial M}{\partial t}+\dfrac{1}{R\,cos\,\theta }\dfrac{\partial }{\partial \lambda }\left( \dfrac{M^2}{D}\right) +\dfrac{1}{R}\dfrac{\partial }{\partial \theta }\left( \dfrac{MN}{D}\right) +&\dfrac{gh}{R\,cos\,\theta }\dfrac{\partial \eta }{\partial \lambda }+\dfrac{g\,n^2}{D^{7/3}}M\sqrt{M^2+N^2}+2\omega \,N\,sin\,\theta \\&-\dfrac{1}{R\,cos\,\theta }\dfrac{\partial }{\partial \lambda } \left[ \dfrac{h^2}{3} \dfrac{1}{R\,cos\,\theta } \left( \dfrac{\partial ^2 M}{\partial \lambda \partial t} + \dfrac{\partial ^2\left( N\,cos\,\theta \right) }{\partial \theta \partial t} \right) \right] =0 \end{aligned} \end{aligned}$$

6 $$\begin{aligned}{}&\begin{aligned} \dfrac{\partial N}{\partial t}+\dfrac{1}{R\,cos\,\theta }\dfrac{\partial }{\partial \lambda }\left( \dfrac{MN}{D}\right) +\dfrac{1}{R}\dfrac{\partial }{\partial \theta }\left( \dfrac{N^2}{D}\right) +&\dfrac{gh}{R}\dfrac{\partial \eta }{\partial \theta }+\dfrac{g\,n^2}{D^{7/3}}N\sqrt{M^2+N^2}-2\omega \,M\,sin\,\theta \\&-\dfrac{1}{R}\dfrac{\partial }{\partial \theta } \left[ \dfrac{h^2}{3} \dfrac{1}{R\,cos\,\theta } \left( \dfrac{\partial ^2 M}{\partial \lambda \partial t} + \dfrac{\partial ^2\left( N\,cos\,\theta \right) }{\partial \theta \partial t} \right) \right] =0 \end{aligned} \end{aligned}$$

where $\eta$ is the water level; *M* and *N* are discharge fluxes in the $\lambda$ (along a parallel of latitude) and $\theta$ (along the longitude cycle) directions, respectively; *D* is the total depth; *g* is the gravitational constant; *R* is the Earth’s radius; *n* is Manning’s roughness coefficient; *h* is the static water depth; and $\omega$ is the angular velocity of Earth’s rotation. In this study, the bottom friction is based on Manning’s roughness coefficient of 0.025^28,60^, and the finite differential equation is solved at each time step of 0.01 seconds^29^. The Open Multi-Processing (OpenMP) platform was adopted to achieve a faster computation time. Along the boundary line, the open sea is limited by nonreflective boundary conditions, and specific conditions for wet/dry fronts must be considered in coastal areas^30,56^. We obtained the tsunami height by using Eq. 4, the continuity equation. The tsunami dispersive effect is caused by the last term on the left-hand side of Eqs. 5 and 6, the momentum equation. In this equation system, it is expected that the dispersion strengthens with increasing water depth (*h*). The momentum equation becomes numerically implicit in time because of the existence of the dispersion term. To solve this system, we adopted the Gauss-Seidel method as an iteration procedure^50^. For computational efficiency, we first solved the derivatives with respect to the time of the tsunami velocity components rather than calculating the tsunami velocity by implicitly solving Eqs. 5 and 6. Integration with respect to time was then explicitly performed by using the estimated derivatives with respect to the time of tsunami velocities^61^.

Topography and bathymetry data for the Tonga Trench and surrounding island areas were provided by the General Bathymetric Chart of the Oceans (GEBCO)^62^, nautical charts, LiDAR topography, multibeam bathymetry data, other offshore surveys, and hand-digitized data. This dataset was compiled as part of a Multi-Hazard Assessment project supported by the Asian Development Bank^63,64^ that was provided through the Tsunami Bulletin Board^65^. The datasets were resampled to four domains with resolutions of 1, 3, 15, and 60 arcseconds, as shown in Fig. 1a. The region of 60 arcseconds was resampled from the GEBCO data to cover the Tonga Trench, and the region of 15 arcseconds was directly used and was cropped to cover only Tonga’s Tongatapu and HTHH volcano areas. Regions of 3 arcseconds and 1 arcsecond were generated by using the cubic spline method in QGIS^11^. The bathymetry and topography data around the HTHH area were digitized based on Brenna et al.^32^, as shown in Fig. 1b,c.

The hypothesized bathymetric change from the eruption explosion of the 2022 HTHH was approximately 200 m below sea level, as shown in Fig. 1b, and spatial shown in Fig. 1c at the volcano. The collapsed surface of the volcano was approximately 3 km in diameter, as estimated by satellite images from 2022 MAXAR Technologies before and after the eruption explosion^5^. Figure 1d shows the volcano before the eruption explosion on 6 January 2022, and Fig. 1e shows the volcano after the eruption explosion on 18 January 2022. The island in the middle of the volcano (red dash cycle in Fig. 1e) disappeared. We used the topographic map to identify the collapsed area due to the eruption explosion.

### Waveform analysis

Spectral analysis reveals oscillation patterns and frequency characteristics^43,66^. In this study, the Fourier transform represented by the fast Fourier transform (FFT) method was applied for spectral analysis, and the adopted FFT is based on the Numpy library in the Python package^67^. The FFT was used to provide the spectral amplitude as a function of frequency (or period), which is widely used in tsunami signal analysis^68–70^. We applied the FFT to the real and synthetic waveform of this event, in which the time series of the waveform was used for 3 hours following the eruption explosion, and we plotted the spectra to present the relationship between the period and amplitude (Supplementary Material 4 and 5).

## Supplementary Information

Supplementary Information 1.

Supplementary Information 2.

Supplementary Information 3.

Supplementary Information 4.

Supplementary Information 5.

Supplementary Information 6.

Supplementary Information 7.

## Data Availability

The tsunami waveform observation data used in this study are available from the GeoNet program of New Zealand’s Institute of Geological and Nuclear Science (GNS) (https://www.geonet.org.nz/tsunami/dart) and SEA LEVEL STATION MONITORING FACILITY (http://www.ioc-sealevelmonitoring.org). The bathymetry data was download from GEBCO (https://www.gebco.net/data_and_products/gridded_bathymetry_data/gebco_30_second_grid). The topography data was download from TBB (https://list.woc.noaa.gov/private/tsunami_bb/2022-February/000236.html).

## Code availability

The code that supports the findings in this study are available fron the corresponding author upon resonable request.
