# Supplementary material for: The near-field tsunami generated by the 15 January 2022 eruption of the Hunga Tonga-Hunga Ha’apai volcano and its impact on Tongatapu, Tonga
Source: Sci Rep. 2022 Sep 7;12:15187. doi: 10.1038/s41598-022-19486-w (PMC9452557; doi:10.1038/s41598-022-19486-w)
Supplement: Supplementary file 7 — Supplementary Information 7. [file 41598_2022_19486_MOESM7_ESM.pdf]

The near-field tsunami generated by the 15 January 2022 eruption of the Hunga Tonga-Hunga Ha'apai volcano and its impact on Tongatapu, Tonga

Supplementary document

KWANCHAI PAKOKSUNG[1,*], ANAWAT SUPPASRI[1], AND FUMIHIKO IMAMURA[1]

[1] *International Research Institute of Disaster Science, Tohoku University, Sendai, 980-0845, Japan*
[*] *pakoksung@irides.tohoku.ac.jp*

Figure and table list

Figure S1 Topgraphy datasets.
Figure S2 Mechanism of initial water displacement.
Table S1 Parameter of initial water level on each candidate explosion energy.
Figure S3 Estimated initial water level based on the explosion energy that varied from 1 to 90 megatons of trinitrotoluene (Mt)
Figure S4 Estimated initial water level on the explosion energy; 1.0 Mt - 12.5 Mt.
Figure S4 (Cont.) Estimated initial water level on the explosion energy; 15.0 Mt - 30.0 Mt.
Figure S4 (Cont.) Estimated initial water level on the explosion energy; 35.0 Mt - 60.0 Mt.
Figure S4 (Cont.) Estimated initial water level on the explosion energy; 75.0 Mt - 90.0 Mt.
Figure S5 Tsunami simulation results for the 1.0 Mt.
Figure S5 (Cont.) Tsunami simulation results for the 2.5 Mt.
Figure S5 (Cont.) Tsunami simulation results for the 5.0 Mt.
Figure S5 (Cont.) Tsunami simulation results for the 7.5 Mt.
Figure S5 (Cont.) Tsunami simulation results for the 10.0 Mt.
Figure S5 (Cont.) Tsunami simulation results for the 12.5 Mt.
Figure S5 (Cont.) Tsunami simulation results for the 15.0 Mt.
Figure S5 (Cont.) Tsunami simulation results for the 17.5 Mt.
Figure S5 (Cont.) Tsunami simulation results for the 20.0 Mt.
Figure S5 (Cont.) Tsunami simulation results for the 22.5 Mt.
Figure S5 (Cont.) Tsunami simulation results for the 25.0 Mt.
Figure S5 (Cont.) Tsunami simulation results for the 30.0 Mt.
Figure S5 (Cont.) Tsunami simulation results for the 35.0 Mt.
Figure S5 (Cont.) Tsunami simulation results for the 40.0 Mt.
Figure S5 (Cont.) Tsunami simulation results for the 45.0 Mt.
Figure S5 (Cont.) Tsunami simulation results for the 50.0 Mt.
Figure S5 (Cont.) Tsunami simulation results for the 55.0 Mt.
Figure S5 (Cont.) Tsunami simulation results for the 60.0 Mt.
Figure S5 (Cont.) Tsunami simulation results for the 75.0 Mt.
Figure S5 (Cont.) Tsunami simulation results for the 90.0 Mt.
Figure S6 Sensitivity of the proposed model.
Figure S7 Comparison of the wave distribution with and without the dispersive effect.
Figure S7 (Cont.) Comparison of the wave distribution with and without the dispersive effect.
Figure S8 Comparison of the waveform with and without the dispersive effect.
Figure S9 Staggered-grid system for the finite difference method for tsunami simulation with nonlinear dispersive eqautions.

Discretization of dispersive momentum equation

Equations 5 and 6 (Momentum equation) in the main text are as follows:

$$\begin{aligned}\frac{\partial M}{\partial t} &+ \frac{1}{R\cos\theta}\frac{\partial}{\partial\lambda}\left(\frac{M^2}{D}\right) + \frac{1}{R}\frac{\partial}{\partial\theta}\left(\frac{MN}{D}\right) + \frac{gh}{R\cos\theta}\frac{\partial\eta}{\partial\lambda} + \frac{g\,n^2}{D^{7/3}}M\sqrt{M^2+N^2}\\ &+2\omega\,N\sin\theta - \frac{1}{R\cos\theta}\frac{\partial}{\partial\lambda}\left[\frac{h^2}{3}\frac{1}{R\cos\theta}\left(\frac{\partial^2 M}{\partial\lambda\partial t} + \frac{\partial^2\left(N\cos\theta\right)}{\partial\theta\partial t}\right)\right] = 0\end{aligned} \tag{S1}$$

$$\begin{aligned}\frac{\partial N}{\partial t} &+ \frac{1}{R\cos\theta}\frac{\partial}{\partial\lambda}\left(\frac{MN}{D}\right) + \frac{1}{R}\frac{\partial}{\partial\theta}\left(\frac{N^2}{D}\right) + \frac{gh}{R}\frac{\partial\eta}{\partial\theta} + \frac{g\,n^2}{D^{7/3}}N\sqrt{M^2+N^2}\\ &-2\omega\,M\sin\theta - \frac{1}{R}\frac{\partial}{\partial\theta}\left[\frac{h^2}{3}\frac{1}{R\cos\theta}\left(\frac{\partial^2 M}{\partial\lambda\partial t} + \frac{\partial^2\left(N\cos\theta\right)}{\partial\theta\partial t}\right)\right] = 0\end{aligned} \tag{S2}$$

The finite difference calculation was performed in the staggered-grid layout as shown in Figure S9. The second-order of the finite-difference scheme of Equations S1 and S2 were discretized in space with following:

$$\begin{aligned}\dot{M}^t_{i,j} =& -\frac{1}{R\cos\theta\Delta\lambda}\left[a_{11}\frac{\left(M^t_{i+1,j}\right)^2}{D\lambda^t_{i+1,j}} + a_{12}\frac{\left(M^t_{i,j}\right)^2}{D\lambda^t_{i,j}} + a_{13}\frac{\left(M^t_{i-1,j}\right)^2}{D\lambda^t_{i-1,j}}\right]\\ &-\frac{1}{R\Delta\theta}\left[b_{11}\frac{M^t_{i,j+1}\cdot\bar{N}^t_{i,j+1}}{D\lambda^t_{i,j+1}} + b_{12}\frac{M^t_{i,j}\cdot\bar{N}^t_{i,j}}{D\lambda^t_{i,j}} + b_{13}\frac{M^t_{i,j-1}\cdot\bar{N}^t_{i,j-1}}{D\lambda^t_{i,j-1}}\right]\\ &-\frac{gh\lambda_{i,j}}{R\cos\theta\Delta\lambda}\cdot\left(\eta^t_{i+1,j}-\eta^t_{i,j}\right) - \frac{gn^2}{\left(D\lambda^t_{i,j}\right)^{7/3}}\cdot M^t_{i,j}\sqrt{\left(M^t_{i,j}\right)^2+\left(\bar{N}^t_{i,j}\right)^2}\\ &-2\omega N^t_{i,j}\sin\theta + \frac{h\lambda^2_{i,j}}{3R^2\cos^2\theta\Delta\lambda^2}\cdot\left(\dot{M}^t_{i+1,j}-2\dot{M}^t_{i,j}+\dot{M}^t_{i-1,j}\right)\\ &+\frac{h\lambda^2_{i,j}}{3R^2\cos^2\theta\Delta\lambda\Delta\theta}\cdot\\ &\left(\dot{N}^t_{i+1,j}\cos\left(\theta\right) - \dot{N}^t_{i+1,j-1}\cos\left(\theta-\Delta\theta\right) - \dot{N}^t_{i,j}\cos\left(\theta\right) + \dot{N}^t_{i,j-1}\cos\left(\theta-\Delta\theta\right)\right)\end{aligned} \tag{S3}$$

$$\begin{aligned}\dot{N}^t_{i,j} =& -\frac{1}{R\cos\theta\Delta\lambda}\left[a_{21}\frac{\bar{M}^t_{i+1,j}\cdot N^t_{i+1,j}}{D\theta^t_{i+1,j}} + a_{22}\frac{\bar{M}^t_{i,j}\cdot N^t_{i,j}}{D\theta^t_{i,j}} + a_{23}\frac{\bar{M}^t_{i-1,j}\cdot N^t_{i-1,j}}{D\theta^t_{i-1,j}}\right]\\ &-\frac{1}{R\Delta\theta}\left[b_{21}\frac{\left(N^t_{i,j+1}\right)^2}{D\theta^t_{i,j+1}} + b_{22}\frac{\left(N^t_{i,j}\right)^2}{D\theta^t_{i,j}} + b_{23}\frac{\left(N^t_{i,j-1}\right)^2}{D\theta^t_{i,j-1}}\right]\\ &-\frac{gh\theta_{i,j}}{R\Delta\theta}\cdot\left(\eta^t_{i,j+1}-\eta^t_{i,j}\right) - \frac{gn^2}{\left(D\theta^t_{i,j}\right)^{7/3}}\cdot N^t_{i,j}\sqrt{\left(\bar{M}^t_{i,j}\right)^2+\left(N^t_{i,j}\right)^2}\\ &+2\omega M^t_{i,j}\sin\theta + \frac{h\theta^2_{i,j}}{3R^2\cos\theta\Delta\lambda\Delta\theta}\cdot\left(\dot{M}^t_{i,j+1}-\dot{M}^t_{i,j}-\dot{M}^t_{i-1,j+1}+\dot{M}^t_{i-1,j}\right)\\ &+\frac{h\theta^2_{i,j}}{3R^2\cos\theta\Delta\theta^2}\cdot\left(\dot{N}^t_{i,j+1}\cos\left(\theta+\Delta\theta\right) - \dot{N}^t_{i,j}\cos\left(\theta\right) + \dot{N}^t_{i,j-1}\cos\left(\theta-\Delta\theta\right)\right)\end{aligned} \tag{S4}$$

where the dots denotes derivatives with respect to time.

We used the Gauss-Seidel method to solve the Eqautions S3 and S4. The derivative with respect to the time of tsunami velocity was updated as follows based on the Eqautions S3 and S4 and an implicit scheme:

$$\begin{aligned}\dot{M}^t_{i,j} =& \frac{3R^2\cos^2\theta\Delta\lambda^2}{3R^2\cos^2\theta\Delta\lambda^2+2h\lambda^2_{i,j}}\cdot Flx^t_{i,j} + \frac{h\lambda^2_{i,j}}{3R^2\cos^2\theta\Delta\lambda^2+2h\lambda^2_{i,j}}\cdot\left(\dot{M}^t_{i+1,j}+\dot{M}^t_{i-1,j}\right)\\ &+\frac{h\lambda^2_{i,j}}{3R^2\cos^2\theta\Delta\lambda^2+2h\lambda^2_{i,j}}\cdot\frac{\Delta\lambda}{\Delta\theta}\cdot\\ &\left(\dot{N}^t_{i+1,j}\cos\left(\theta\right) - \dot{N}^t_{i+1,j-1}\cos\left(\theta-\Delta\theta\right) - \dot{N}^t_{i,j}\cos\left(\theta\right) + \dot{N}^t_{i,j-1}\cos\left(\theta-\Delta\theta\right)\right)\end{aligned} \tag{S5}$$

$$\dot{N}_{i,j}^{t} = \frac{3R^2\Delta\theta^2}{3R^2\Delta\theta^2 + 2h\theta_{i,j}^2} \cdot Fly_{i,j}^{t} + \frac{h\theta_{i,j}^2}{3R^2\Delta\theta^2 + 2h\theta_{i,j}^2} \cdot \frac{\Delta\theta}{cos\ (\theta)\,\Delta\lambda} \cdot \left(\dot{M}_{i,j+1}^{t} - \dot{M}_{i,j}^{t} - \dot{M}_{i-1,j+1}^{t} + \dot{M}_{i-1,j}^{t}\right) + \frac{h\theta_{i,j}^2}{3R^2\Delta\theta^2 + 2h\theta_{i,j}^2} \cdot \left(\dot{N}_{i,j+1}^{t}\, cos\ (\theta + \Delta\theta) + \dot{N}_{i,j-1}^{t}\, cos\ (\theta - \Delta\theta)\right) \tag{S6}$$

Equations S5 and S6 were repeatedly applied until $\dot{M}_{i,j}^{t}$ and $\dot{N}_{i,j}^{t}$ converged. Tsunami velocities in the interior region were then updated by numerical integration with respect to the time as follows:

$$M_{i,j}^{t+1} = M_{i,j}^{t} + \dot{M}_{i,j}^{t} \cdot \Delta t \tag{S7}$$

$$N_{i,j}^{t+1} = N_{i,j}^{t} + \dot{N}_{i,j}^{t} \cdot \Delta t \tag{S8}$$

where

$$Flx_{i,j}^{t} = -\frac{1}{R\,cos\,\theta\Delta\lambda}\left[a_{11}\frac{\left(M_{i+1,j}^{t}\right)^2}{D\lambda_{i+1,j}^{t}} + a_{12}\frac{\left(M_{i,j}^{t}\right)^2}{D\lambda_{i,j}^{t}} + a_{13}\frac{\left(M_{i-1,j}^{t}\right)^2}{D\lambda_{i-1,j}^{t}}\right] - \frac{1}{R\Delta\theta}\left[b_{11}\frac{M_{i,j+1}^{t}\cdot\bar{N}_{i,j+1}^{t}}{D\lambda_{i,j+1}^{t}} + b_{12}\frac{M_{i,j}^{t}\cdot\bar{N}_{i,j}^{t}}{D\lambda_{i,j}^{t}} + b_{13}\frac{M_{i,j-1}^{t}\cdot\bar{N}_{i,j-1}^{t}}{D\lambda_{i,j-1}^{t}}\right] - \frac{gh_{i,j}}{R\,cos\,\theta\Delta\lambda}\cdot\left(\eta_{i+1,j}^{t} - \eta_{i,j}^{t}\right) - \frac{gn^2}{\left(D\lambda_{i,j}^{t}\right)^{7/3}}\cdot M_{i,j}^{t}\sqrt{\left(M_{i,j}^{t}\right)^2 + \left(\bar{N}_{i,j}^{t}\right)^2} - 2\omega N_{i,j}^{t}\,sin\,\theta \tag{S9}$$

$$Fly_{i,j}^{t} = -\frac{1}{R\,cos\,\theta\Delta\lambda}\left[a_{21}\frac{\bar{M}_{i+1,j}^{t}\cdot N_{i+1,j}^{t}}{D\theta_{i+1,j}^{t}} + a_{22}\frac{\bar{M}_{i,j}^{t}\cdot N_{i,j}^{t}}{D\theta_{i,j}^{t}} + a_{13}\frac{\bar{M}_{i-1,j}^{t}\cdot N_{i-1,j}^{t}}{D\theta_{i-1,j}^{t}}\right] - \frac{1}{R\Delta\theta}\left[b_{21}\frac{\left(N_{i,j+1}^{t}\right)^2}{D\theta_{i,j+1}^{t}} + b_{22}\frac{\left(N_{i,j}^{t}\right)^2}{D\theta_{i,j}^{t}} + b_{23}\frac{\left(N_{i,j-1}^{t}\right)^2}{D_{i,j-1}^{t}}\right] - \frac{gh_{i,j}}{R\Delta\theta}\cdot\left(\eta_{i,j+1}^{t} - \eta_{i,j}^{t}\right) - \frac{gn^2}{\left(D\theta_{i,j}^{t}\right)^{7/3}}\cdot N_{i,j}^{t}\sqrt{\left(\bar{M}_{i,j}^{t}\right)^2 + \left(N_{i,j}^{t}\right)^2} + 2\omega M_{i,j}^{t}\,sin\,\theta \tag{S10}$$

$$\begin{aligned} M_{i,j}^{t} &\geq 0, a_{11} = 0, a_{12} = 1, a_{13} = -1 \\ M_{i,j}^{t} &< 0, a_{11} = 1, a_{12} = -1, a_{13} = 0 \\ \bar{N}_{i,j}^{t} &\geq 0, b_{11} = 0, b_{12} = 1, b_{13} = -1 \\ \bar{N}_{i,j}^{t} &< 0, b_{11} = 1, b_{12} = -1, b_{13} = 0 \end{aligned} \tag{S11}$$

$$\begin{aligned} \bar{M}_{i,j}^{t} &\geq 0, a_{21} = 0, a_{22} = 1, a_{23} = -1 \\ \bar{M}_{i,j}^{t} &< 0, a_{21} = 1, a_{22} = -1, a_{23} = 0 \\ N_{i,j}^{t} &\geq 0, b_{21} = 0, b_{22} = 1, b_{23} = -1 \\ N_{i,j}^{t} &< 0, b_{21} = 1, b_{22} = -1, b_{23} = 0 \end{aligned} \tag{S12}$$

$$\begin{aligned} \bar{N}_{i,j} &= \frac{N_{i,j} + N_{i+1,j} + N_{i+1,j-1} + N_{i,j-1}}{4} \\ \bar{M}_{i,j} &= \frac{M_{i-1,j+1} + M_{i,j+1} + M_{i,j} + M_{i-1,j}}{4} \end{aligned} \tag{S13}$$

$$\begin{aligned} D\lambda_{i,j}^{t} &= \frac{D_{i+1,j}^{t} + D_{i,j}^{t}}{2} \\ D\theta_{i,j}^{t} &= \frac{D_{i,j+1}^{t} + D_{i,j}^{t}}{2} \end{aligned} \tag{S14}$$

$$
\begin{aligned}
h\lambda_{i,j} &= \frac{h_{i+1,j} + h_{i,j}}{2} \\
h\theta_{i,j} &= \frac{h_{i,j+1} + h_{i,j}}{2}
\end{aligned}
\tag{S15}
$$

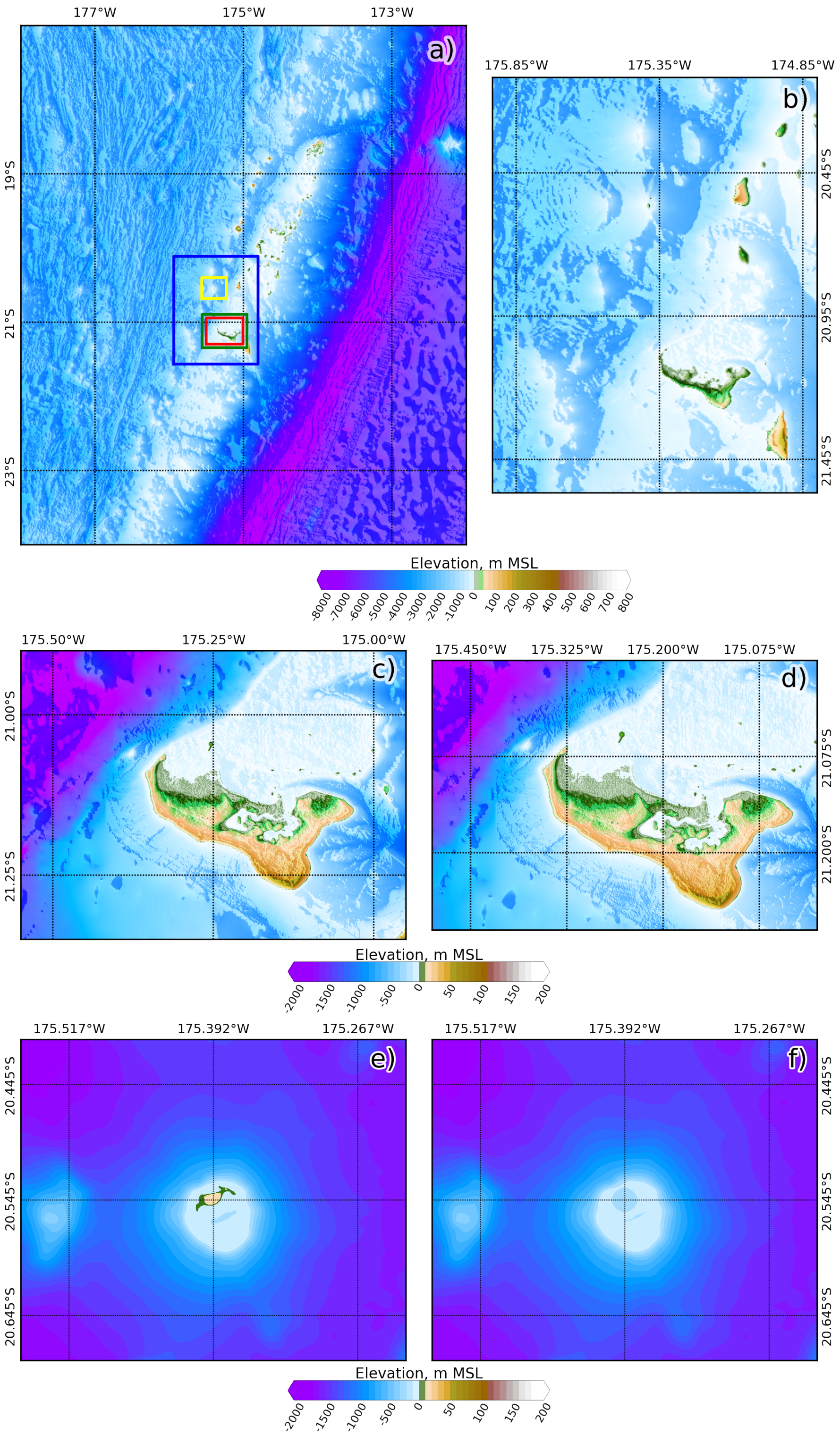

Fig. S1. Topography datasets, a) Region 1st based on the resolution of 60 arcseconds in which, the blue box is 2nd region, the green box is 3rd region, the red box is 4th region and the yellow box is volcano area. b) The region 2nd is based on the resolution of 15 arcseconds. c) The region 3rd is based on the resolution of 3 arcseconds. d) The region 4th is based on the resolution of 1 arcsecond. e) The topography of the volcano area was presented before the 2022 HTHH explosion event and is based on the resolution of 1 arcsecond. f) The hypothesized topography of the volcano area was presented after the 2022 HTHH explosion event and is based on the resolution of 1 arcsecond.

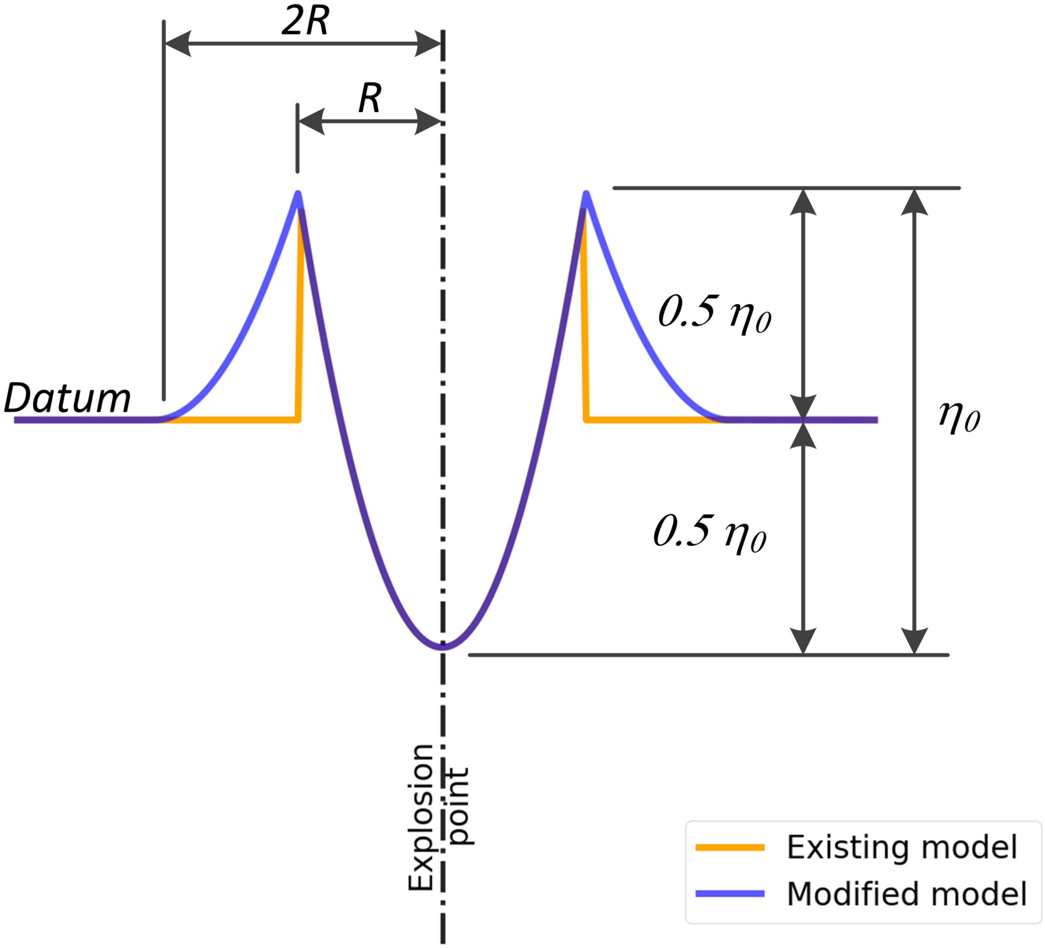

Fig. S2. Mechanism of initial water displacement from subaqueous volcanic explosion, R : diameter of explosion, η_0: maximum water surface displacement.

Table S1. Parameter of initial water level on each candidate explosion energy

E, Mt	R, m	η_0, m (deep sea depth condition)	η_0, m (shallow sea depth condition)
1.0	437.54	80.264	163.334
2.5	593.83	100.006	203.508
5.0	748.18	118.106	240.342
7.5	856.45	130.177	264.906
10.0	942.65	139.483	283.843
12.5	1,015.44	147.156	299.458
15.0	1,079.06	153.738	312.852
17.5	1,135.96	159.533	324.643
20.0	1,187.66	164.728	335.216
22.5	1,235.22	169.451	344.827
25.0	1,279.37	173.791	353.648
30.0	1,359.54	181.546	369.476
35.0	1,431.22	188.407	383.412
40.0	1,496.36	194.543	395.888
45.0	1,556.28	200.121	407.238
50.0	1,611.91	205.245	417.667
55.0	1,663.94	209.994	427.331
60.0	1,712.91	214.426	436.349
75.0	1,845.17	226.222	460.354
90.0	1,960.79	236.341	480.945

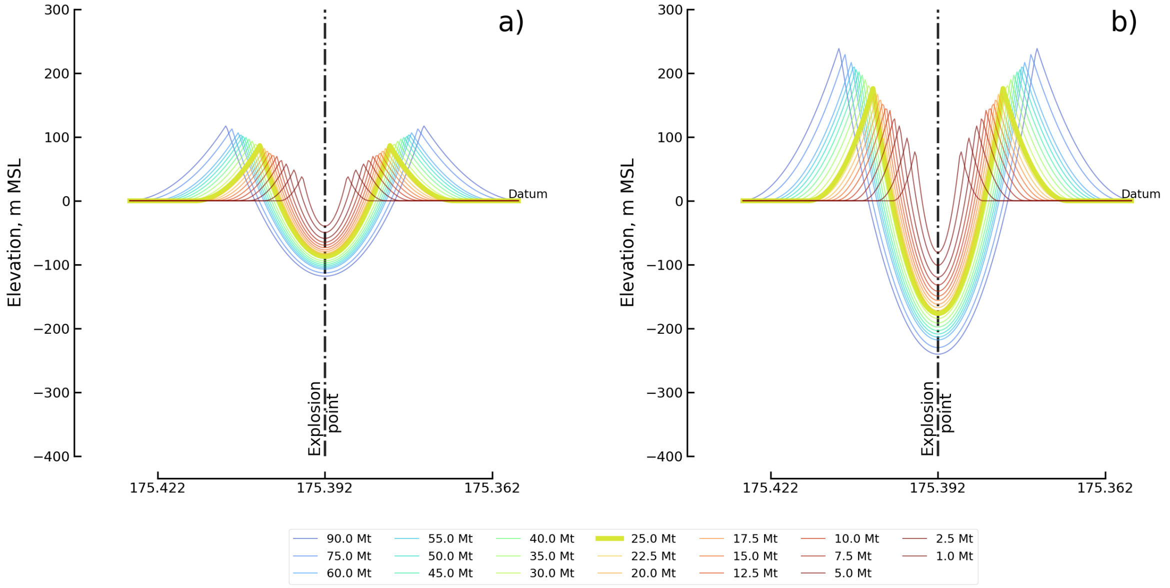

Fig. S3. Estimated initial water level based on the explosion energy that varied from 1 to 90 megatons of trinitrotoluene (Mt); a) The deep sea depth condition, and b) The shallow sea depth condition

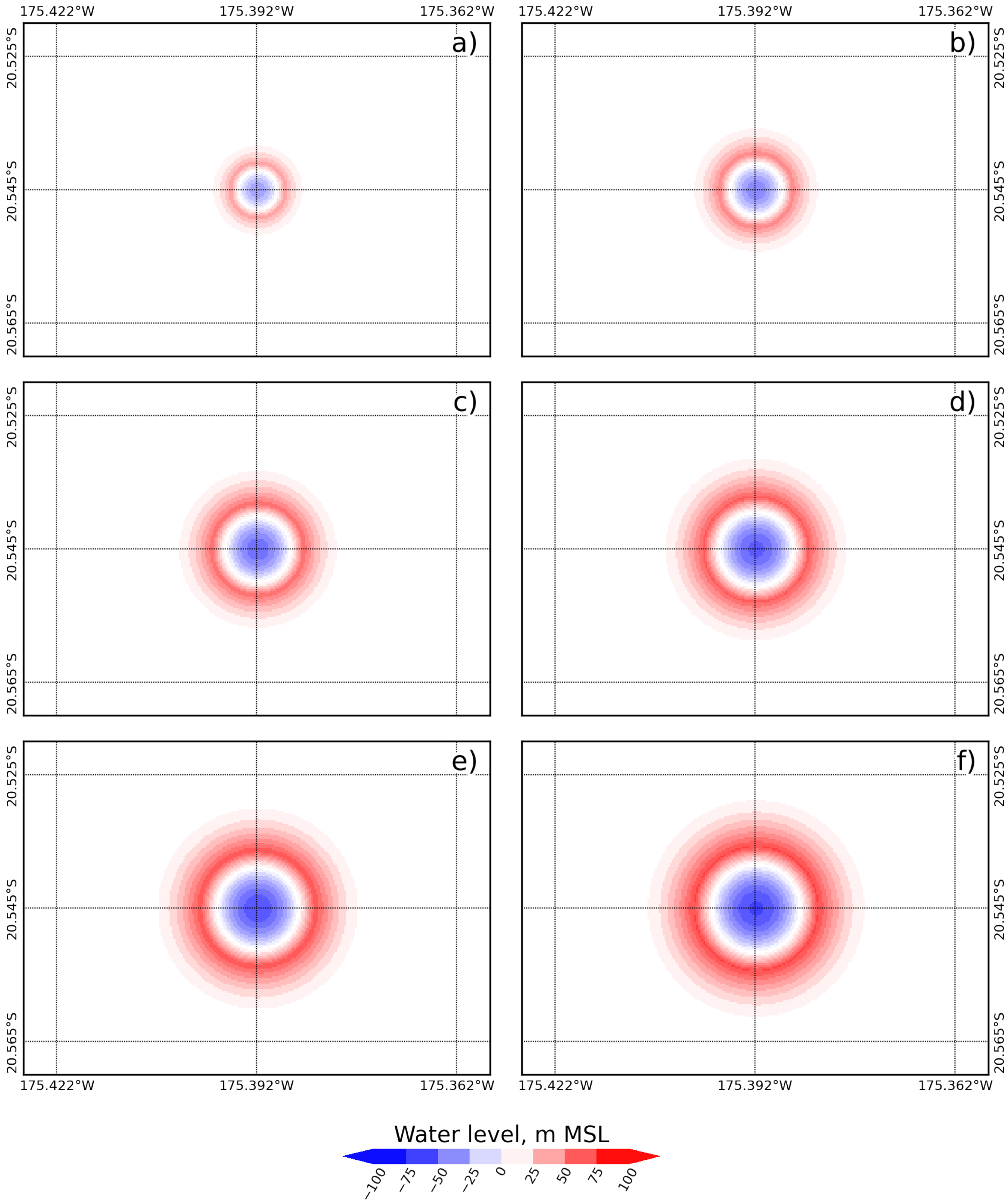

Fig. S4. Estimated initial water level with the deep sea depth condition; a) 1.0 Mt, b) 2.5 Mt , c) 5.0 Mt, d) 7.5 Mt, e) 10.0 Mt, and f) 12.5 Mt

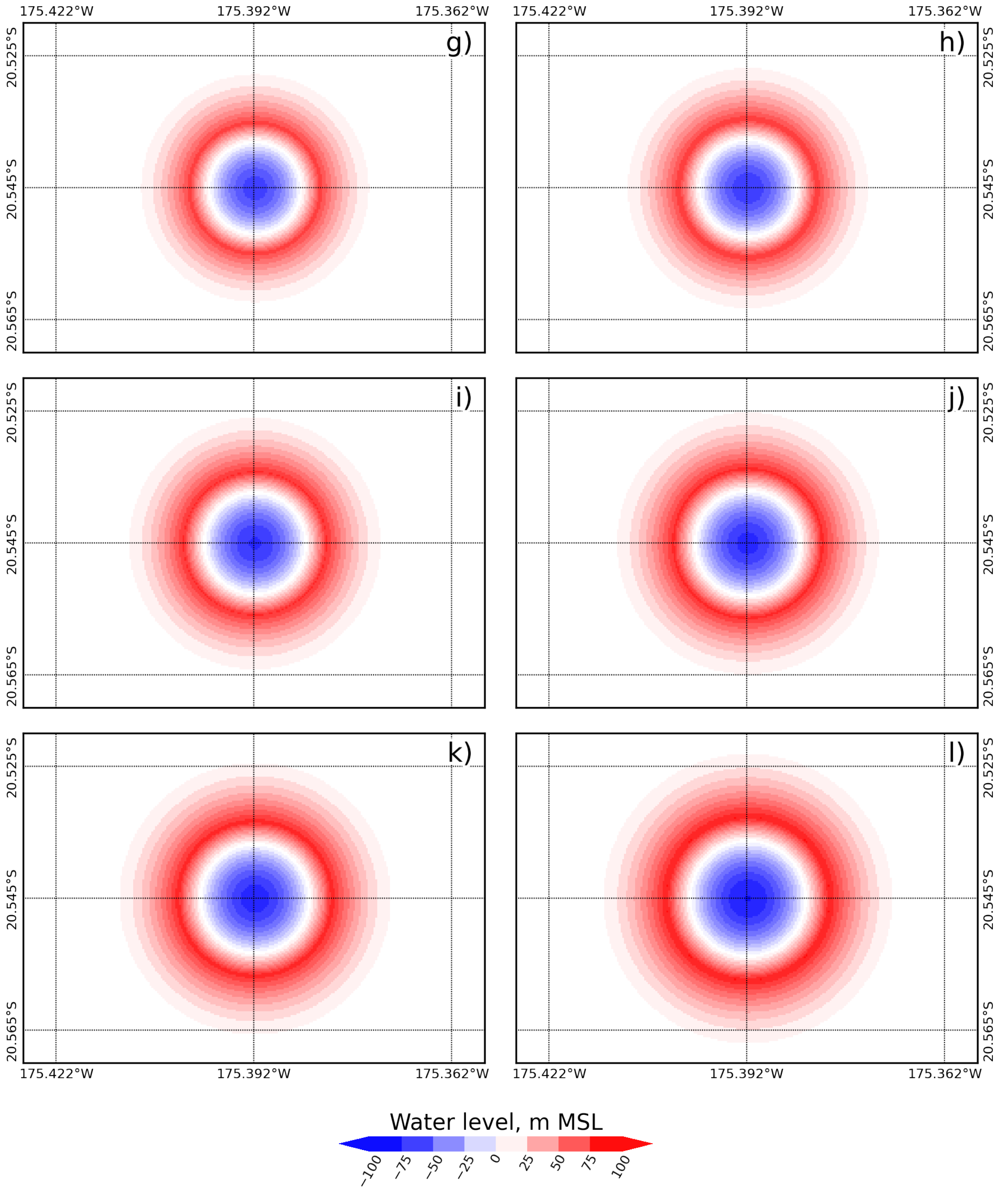

Fig. S4 (Cont.). Estimated initial water level with the deep sea depth condition; g) 15.0 Mt, h) 17.5 Mt , i) 20.0 Mt, j) 22.5 Mt, k) 25.0 Mt, and l) 30.0 Mt

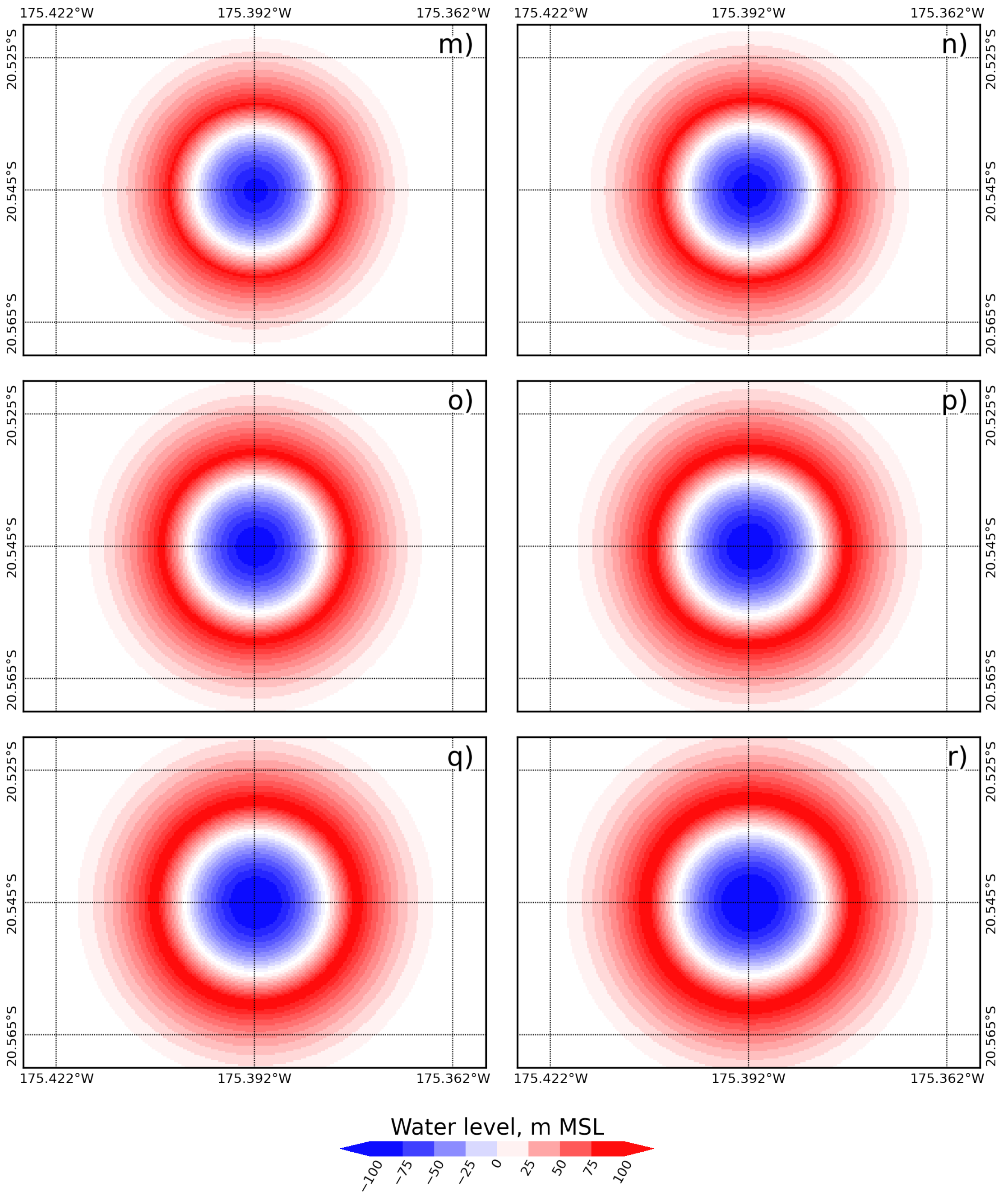

Fig. S4 (Cont.). Estimated initial water level with the deep sea depth condition; m) 35.0 Mt, n) 40.0 Mt , o) 45.0 Mt, p) 50.0 Mt, q) 55.0 Mt, and r) 60.0 Mt

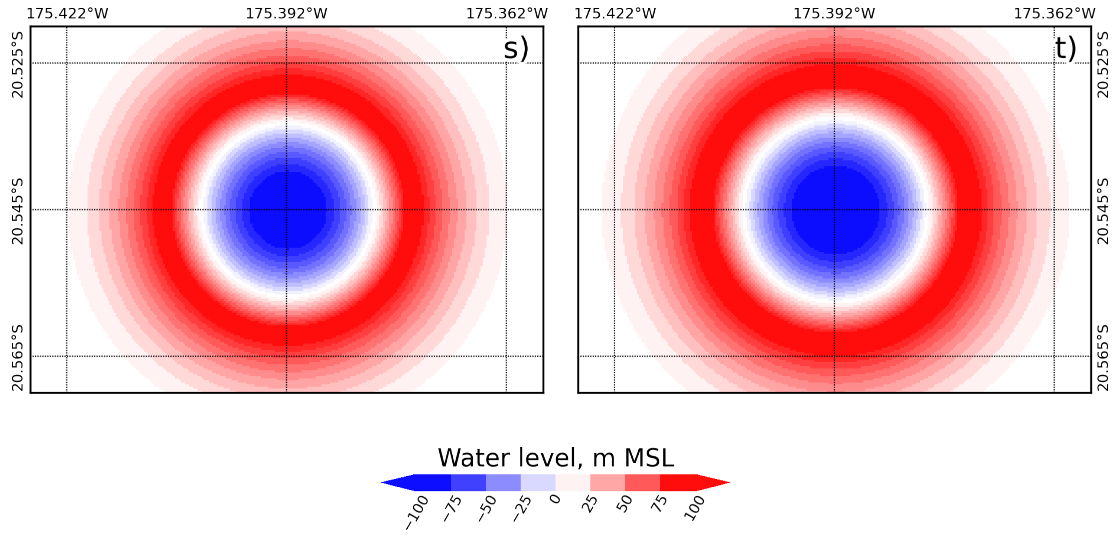

Fig. S4 (Cont.). Estimated initial water level with the deep sea depth condition; s) 75.0 Mt, and t) 90.0 Mt

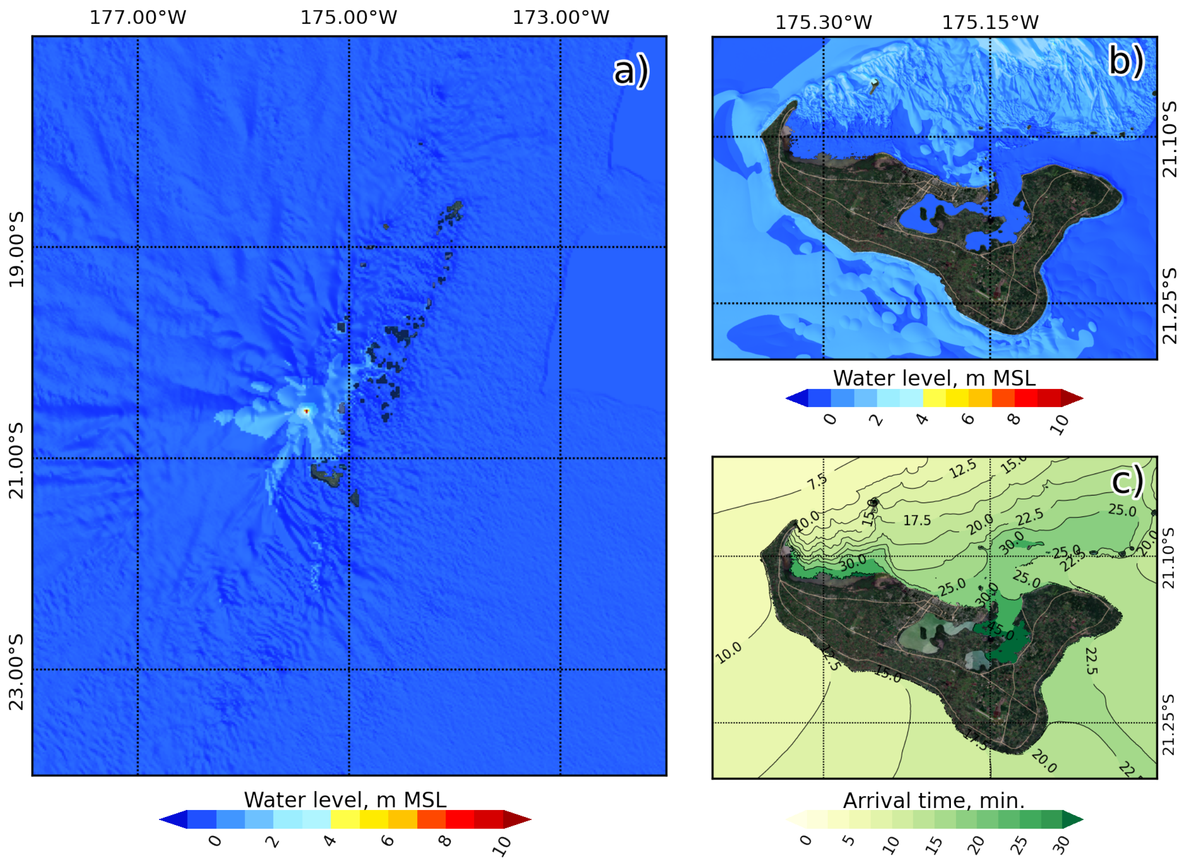

Fig. S5. Tsunami simulation results for the 1.0 Mt with the deep sea depth condition; a) Maximum water level on 1st region, b) Maximum water level on 3rd region, and c) Arrival time on 3rd region. The map was created with a QGIS software, version 3.16.15-Hannover (http://www.qgis.org), and the satellite image for basemap was downloaded from QuickMapServices plugin (https://github.com/nextgis/quickmapservices) through the QGIS.

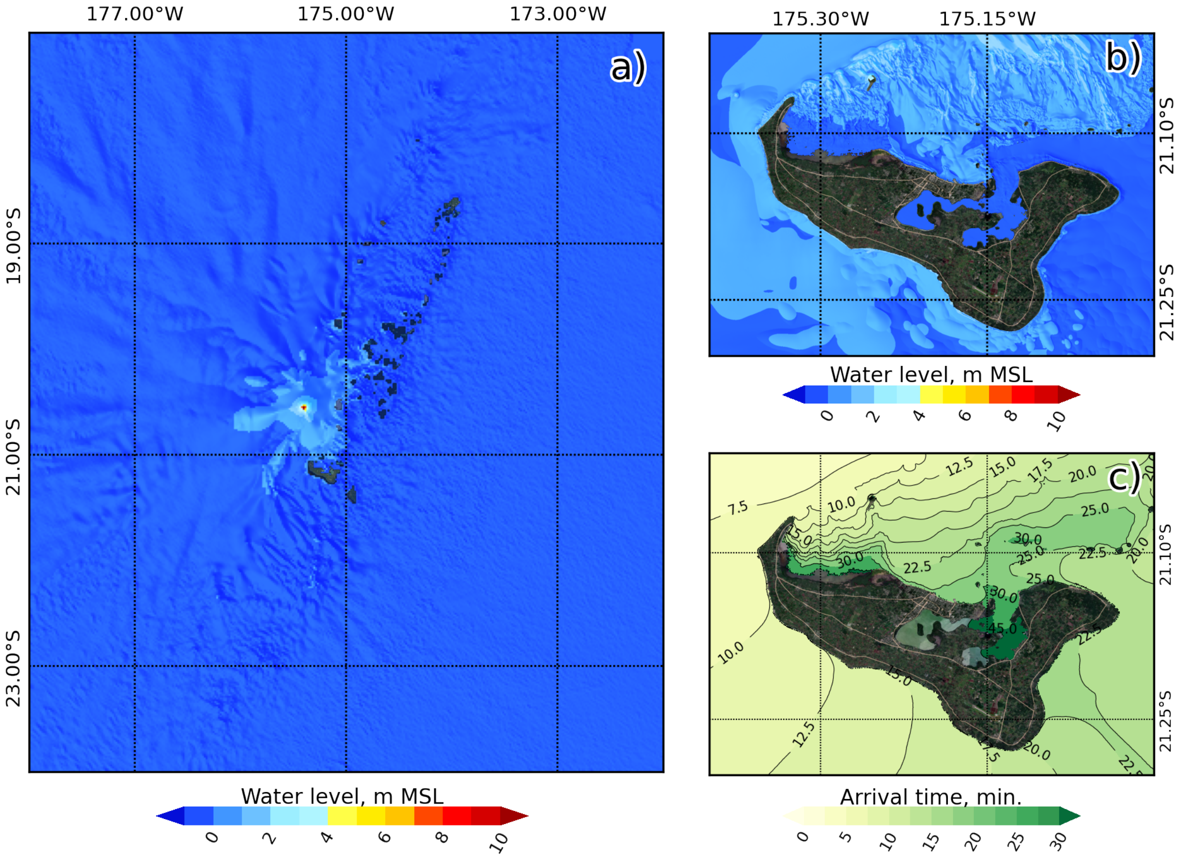

Fig. S5 (Cont.). Tsunami simulation results for the 2.5 Mt with the deep sea depth condition; a) Maximum water level on 1st region, b) Maximum water level on 3rd region, and c) Arrival time on 3rd region. The map was created with a QGIS software, version 3.16.15-Hannover (http://www.qgis.org), and the satellite image for basemap was downloaded from QuickMapServices plugin (https://github.com/nextgis/quickmapservices) through the QGIS.

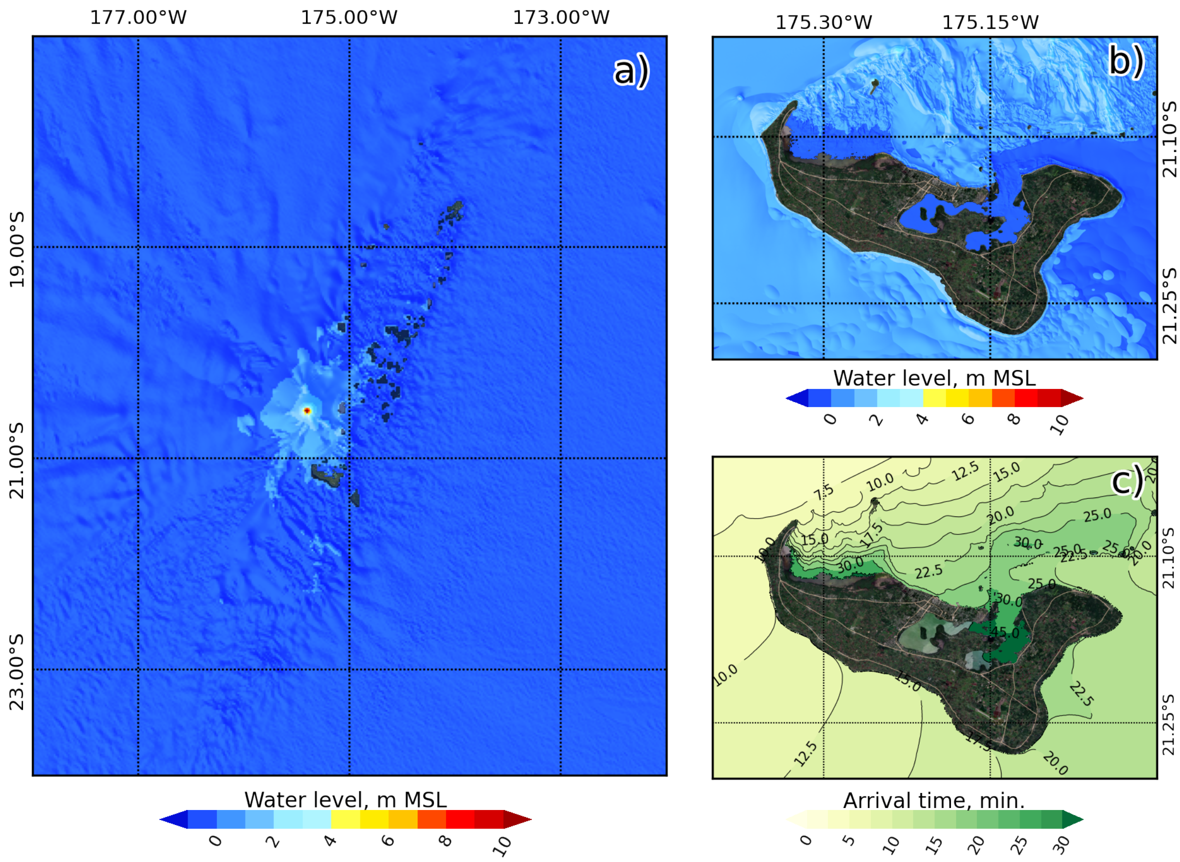

Fig. S5 (Cont.). Tsunami simulation results for the 5.0 Mt with the deep sea depth condition; a) Maximum water level on 1st region, b) Maximum water level on 3rd region, and c) Arrival time on 3rd region. The map was created with a QGIS software, version 3.16.15-Hannover (http://www.qgis.org), and the satellite image for basemap was downloaded from QuickMapServices plugin (https://github.com/nextgis/quickmapservices) through the QGIS.

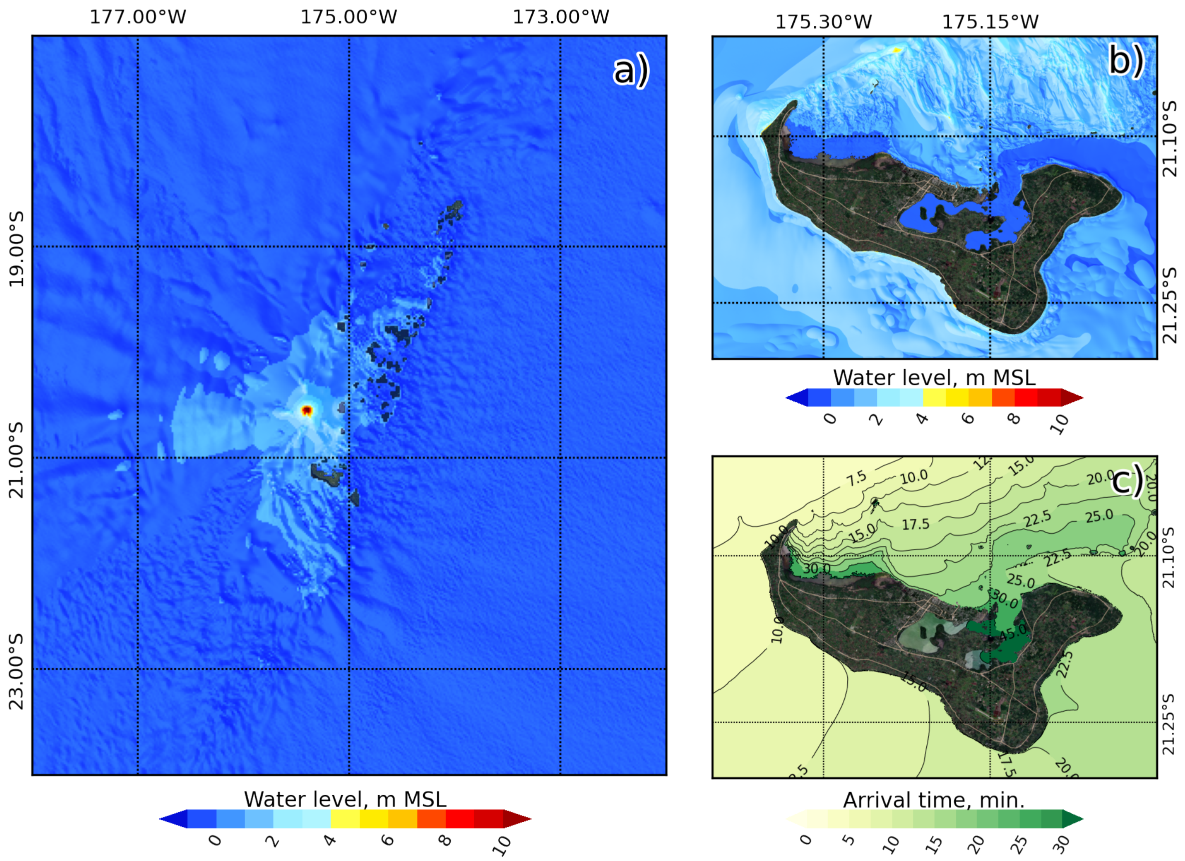

Fig. S5 (Cont.). Tsunami simulation results for the 7.5 Mt with the deep sea depth condition; a) Maximum water level on 1st region, b) Maximum water level on 3rd region, and c) Arrival time on 3rd region. The map was created with a QGIS software, version 3.16.15-Hannover (http://www.qgis.org), and the satellite image for basemap was downloaded from QuickMapServices plugin (https://github.com/nextgis/quickmapservices) through the QGIS.

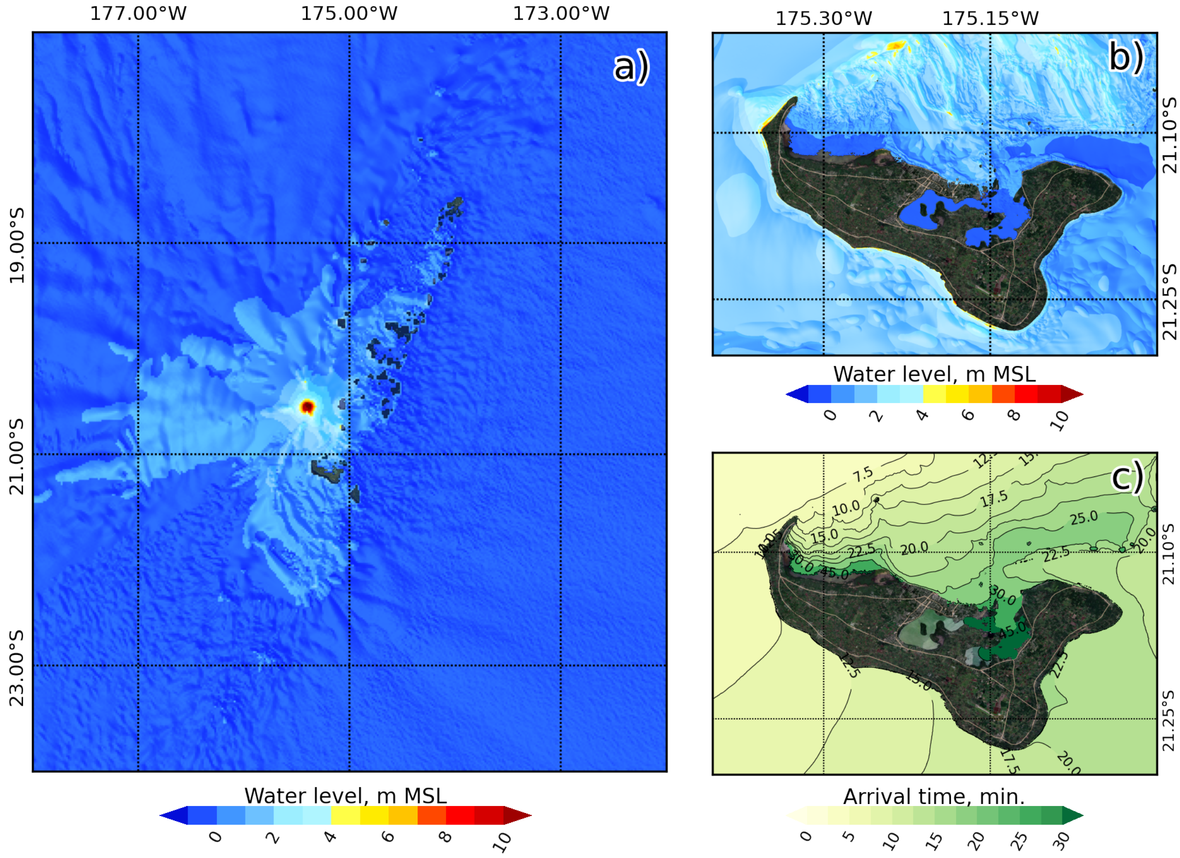

Fig. S5 (Cont.). Tsunami simulation results for the 10.0 Mt with the deep sea depth condition; a) Maximum water level on 1st region, b) Maximum water level on 3rd region, and c) Arrival time on 3rd region. The map was created with a QGIS software, version 3.16.15-Hannover (http://www.qgis.org), and the satellite image for basemap was downloaded from QuickMapServices plugin (https://github.com/nextgis/quickmapservices) through the QGIS.

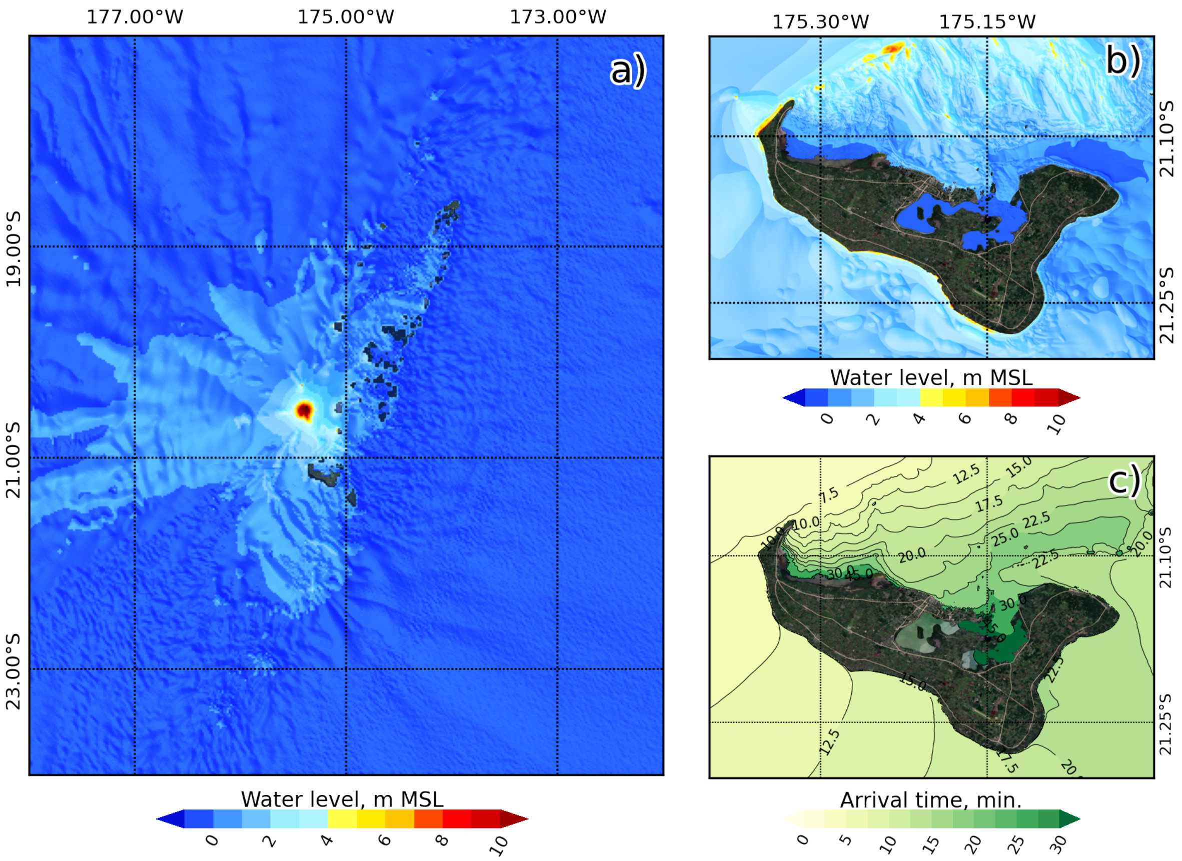

Fig. S5 (Cont.). Tsunami simulation results for the 12.5 Mt with the deep sea depth condition; a) Maximum water level on 1st region, b) Maximum water level on 3rd region, and c) Arrival time on 3rd region. The map was created with a QGIS software, version 3.16.15-Hannover (http://www.qgis.org), and the satellite image for basemap was downloaded from QuickMapServices plugin (https://github.com/nextgis/quickmapservices) through the QGIS.

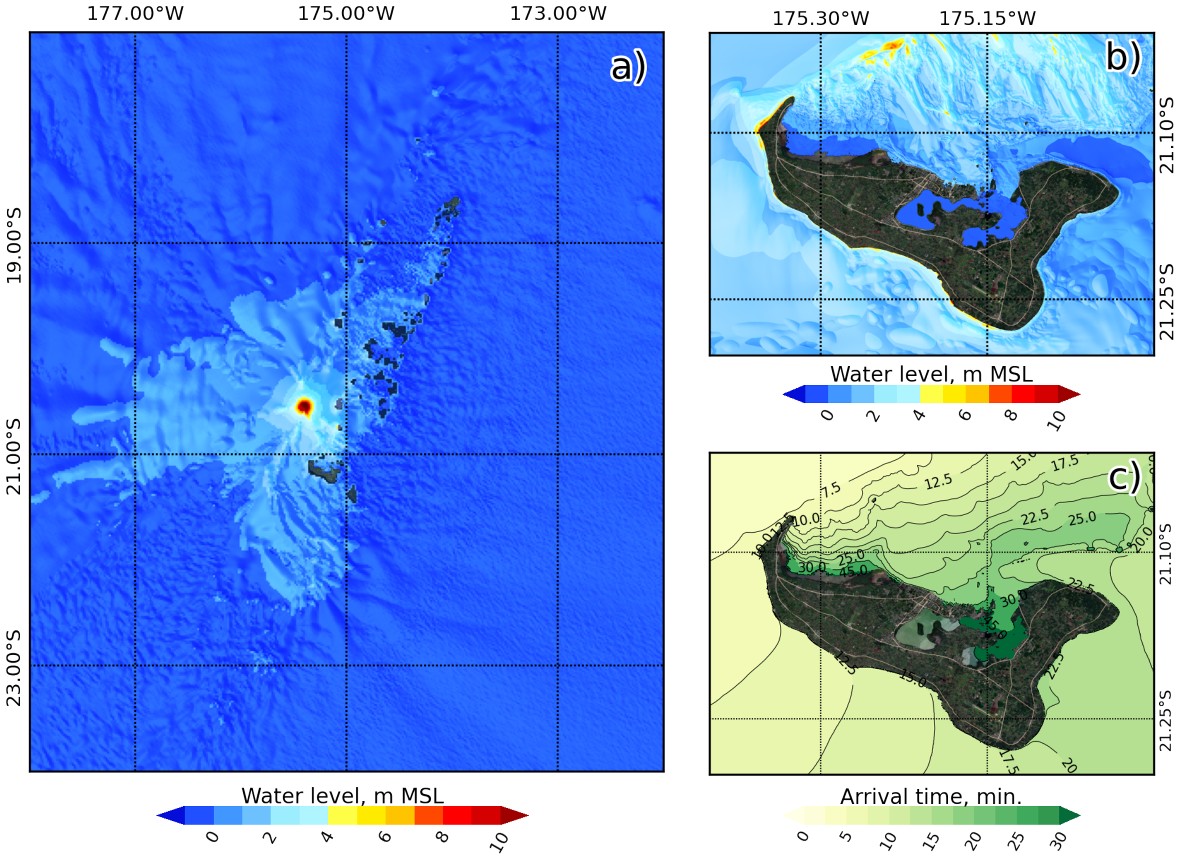

Fig. S5 (Cont.). Tsunami simulation results for the 15.0 Mt with the deep sea depth condition; a) Maximum water level on 1st region, b) Maximum water level on 3rd region, and c) Arrival time on 3rd region. The map was created with a QGIS software, version 3.16.15-Hannover (http://www.qgis.org), and the satellite image for basemap was downloaded from QuickMapServices plugin (https://github.com/nextgis/quickmapservices) through the QGIS.

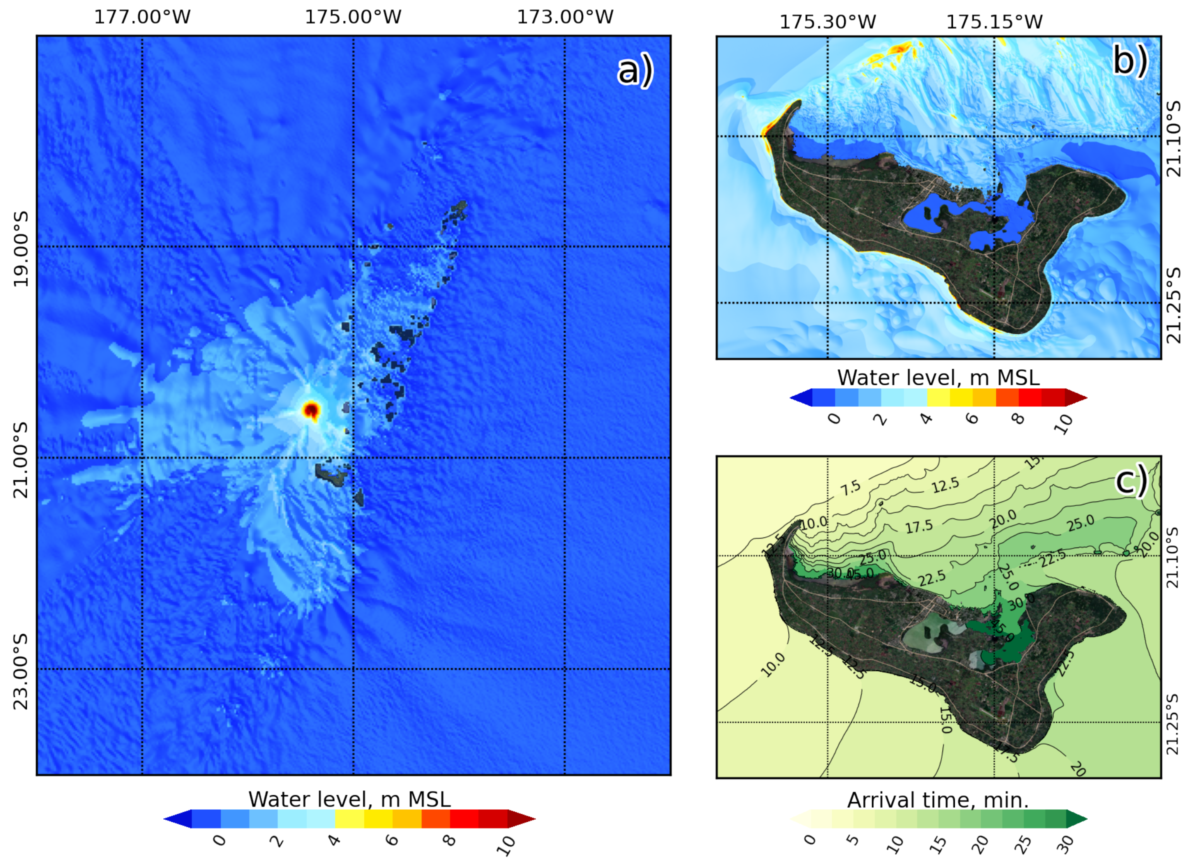

Fig. S5 (Cont.). Tsunami simulation results for the 17.5 Mt with the deep sea depth condition; a) Maximum water level on 1st region, b) Maximum water level on 3rd region, and c) Arrival time on 3rd region. The map was created with a QGIS software, version 3.16.15-Hannover (http://www.qgis.org), and the satellite image for basemap was downloaded from QuickMapServices plugin (https://github.com/nextgis/quickmapservices) through the QGIS.

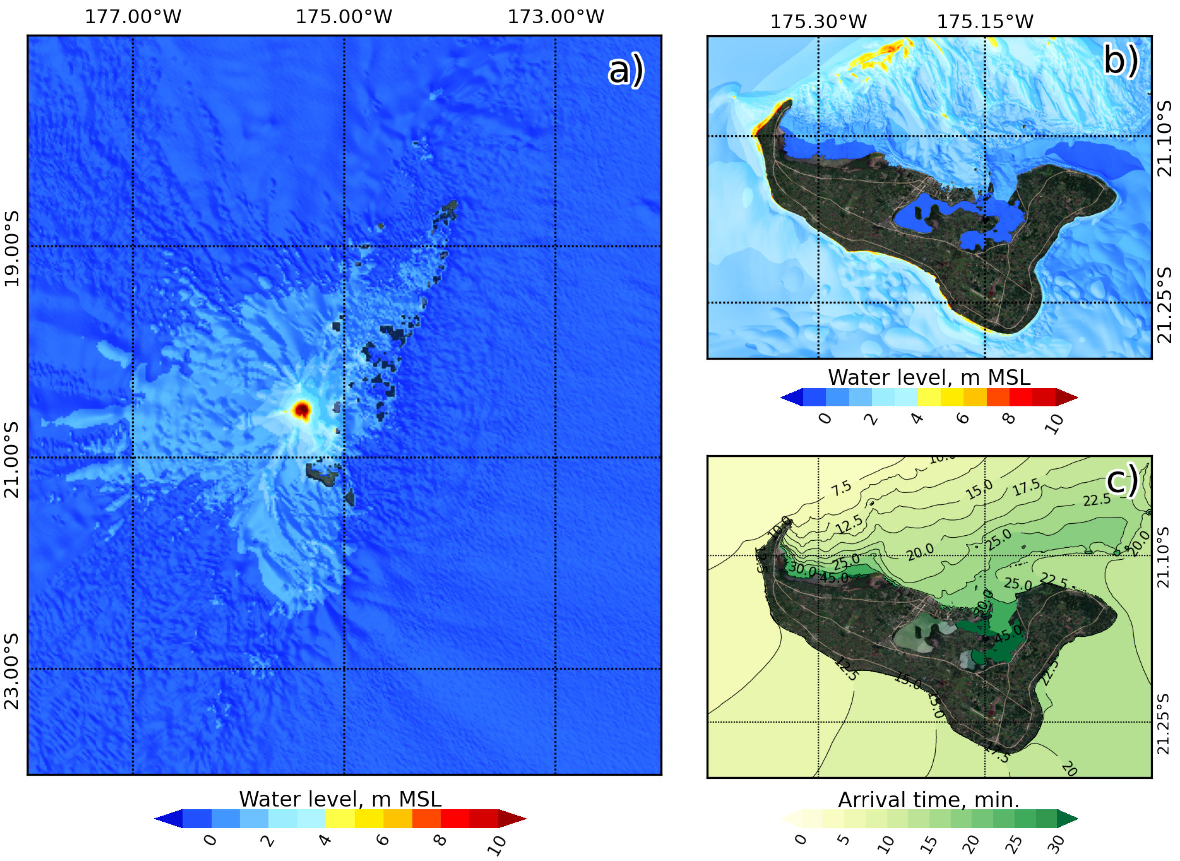

Fig. S5 (Cont.). Tsunami simulation results for the 20.0 Mt with the deep sea depth condition; a) Maximum water level on 1st region, b) Maximum water level on 3rd region, and c) Arrival time on 3rd region. The map was created with a QGIS software, version 3.16.15-Hannover (http://www.qgis.org), and the satellite image for basemap was downloaded from QuickMapServices plugin (https://github.com/nextgis/quickmapservices) through the QGIS[**?**].

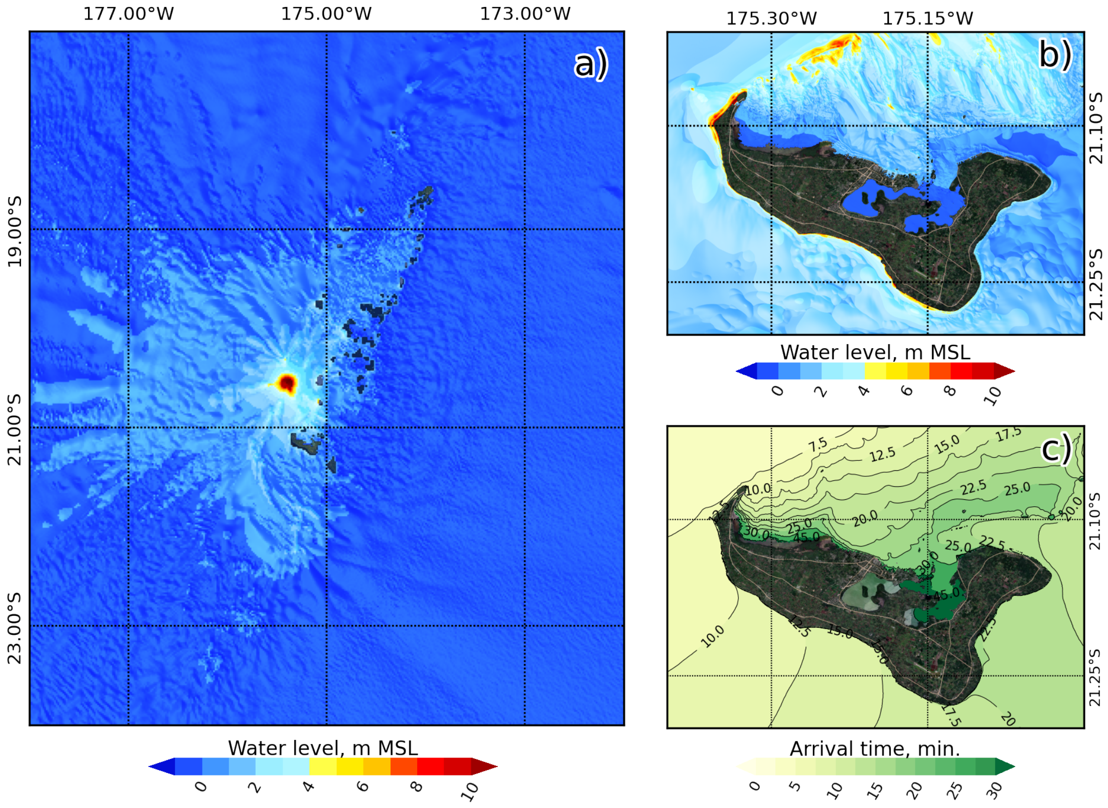

Fig. S5 (Cont.). Tsunami simulation results for the 22.5 Mt with the deep sea depth condition; a) Maximum water level on 1st region, b) Maximum water level on 3rd region, and c) Arrival time on 3rd region. The map was created with a QGIS software, version 3.16.15-Hannover (http://www.qgis.org), and the satellite basemap was downloaded from QuickMapServices plugin (https://github.com/nextgis/quickmapservices) through the QGIS.

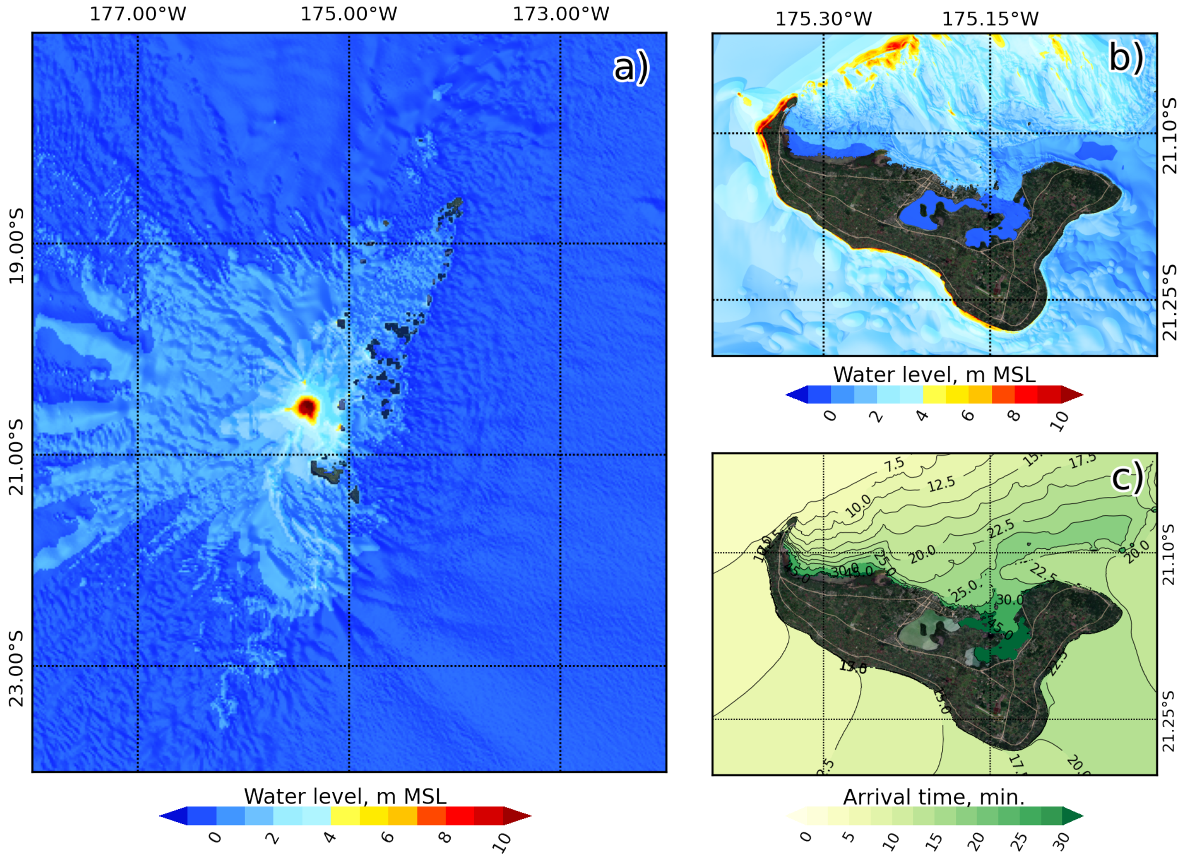

Fig. S5 (Cont.). Tsunami simulation results for the 25.0 Mt with the deep sea depth condition; a) Maximum water level on 1st region, b) Maximum water level on 3rd region, and c) Arrival time on 3rd region. The map was created with a QGIS software, version 3.16.15-Hannover (http://www.qgis.org), and the satellite image for basemap was downloaded from QuickMapServices plugin (https://github.com/nextgis/quickmapservices) through the QGIS.

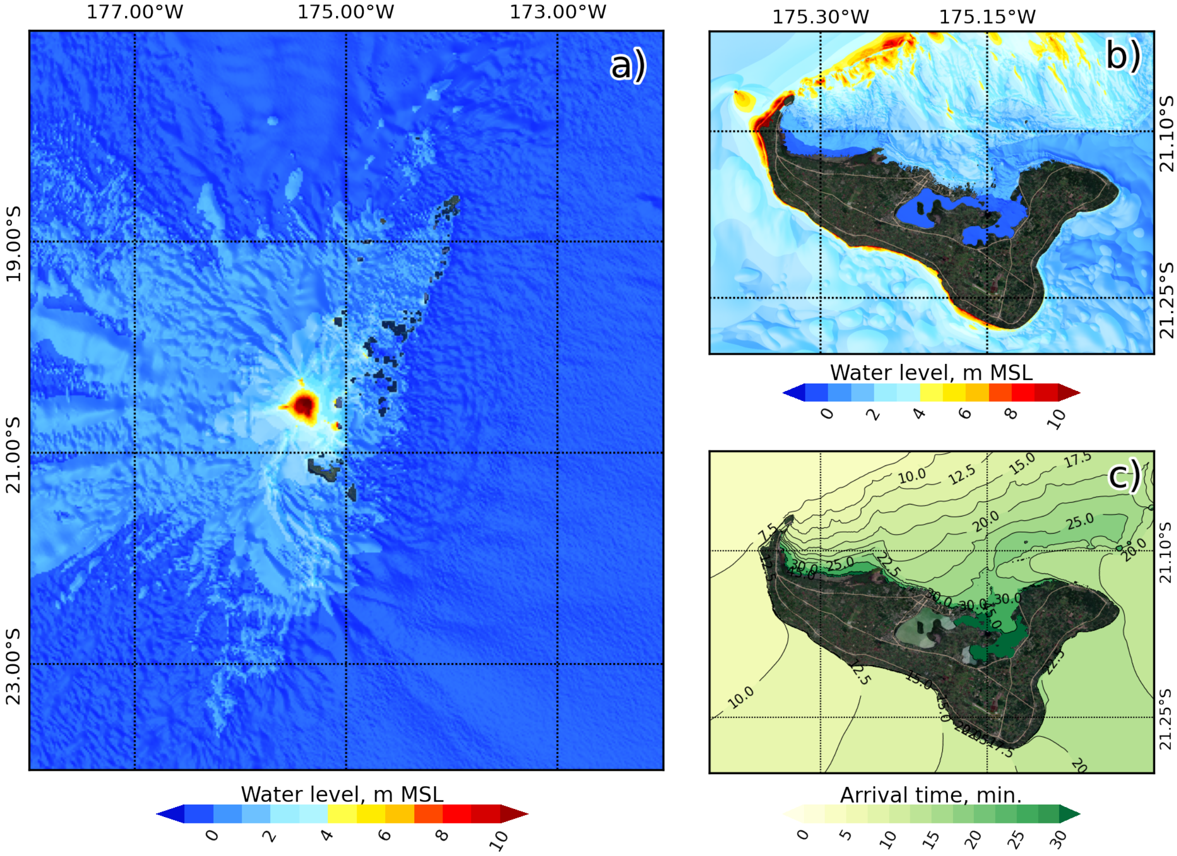

Fig. S5 (Cont.). Tsunami simulation results for the 30.0 Mt with the deep sea depth condition; a) Maximum water level on 1st region, b) Maximum water level on 3rd region, and c) Arrival time on 3rd region. The map was created with a QGIS software, version 3.16.15-Hannover (http://www.qgis.org), and the satellite image for basemap was downloaded from QuickMapServices plugin (https://github.com/nextgis/quickmapservices) through the QGIS.

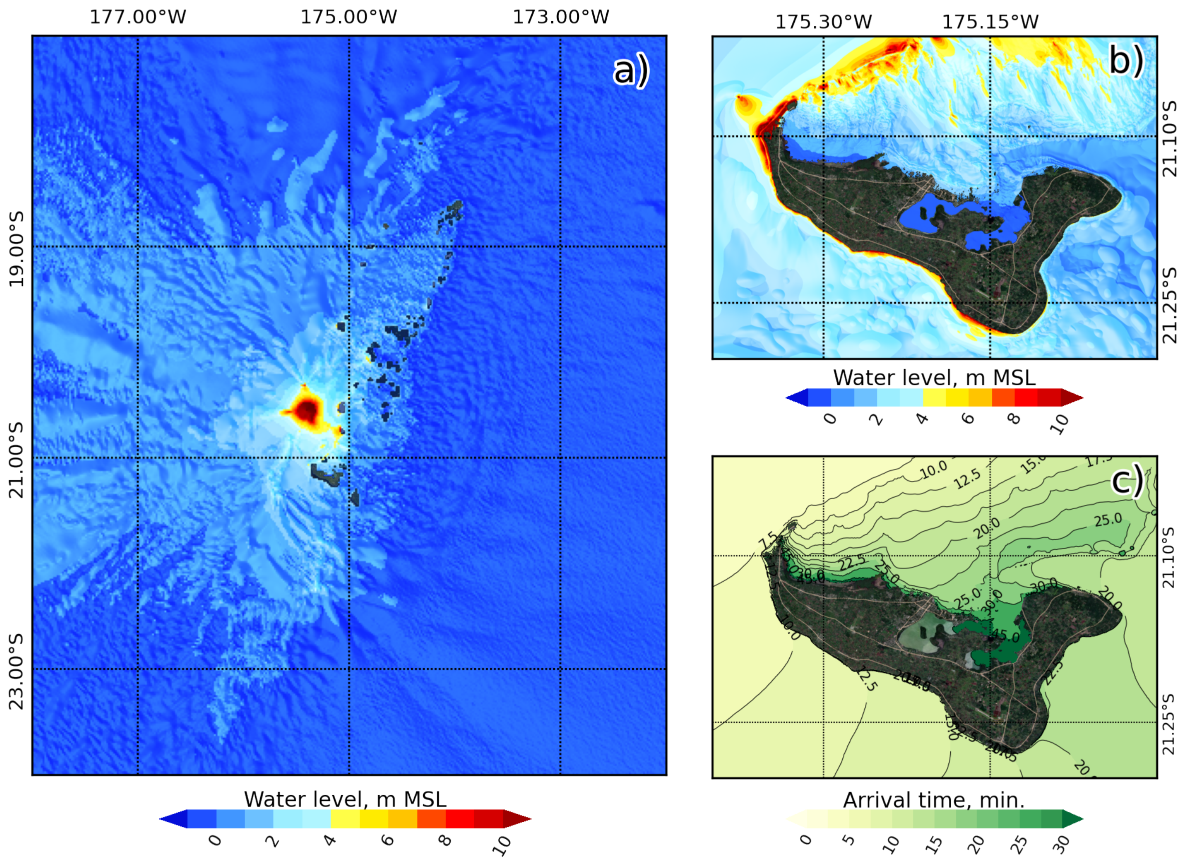

Fig. S5 (Cont.). Tsunami simulation results for the 35.0 Mt with the deep sea depth condition; a) Maximum water level on 1st region, b) Maximum water level on 3rd region, and c) Arrival time on 3rd region. The map was created with a QGIS software, version 3.16.15-Hannover (http://www.qgis.org), and the satellite image for basemap was downloaded from QuickMapServices plugin (https://github.com/nextgis/quickmapservices) through the QGIS.

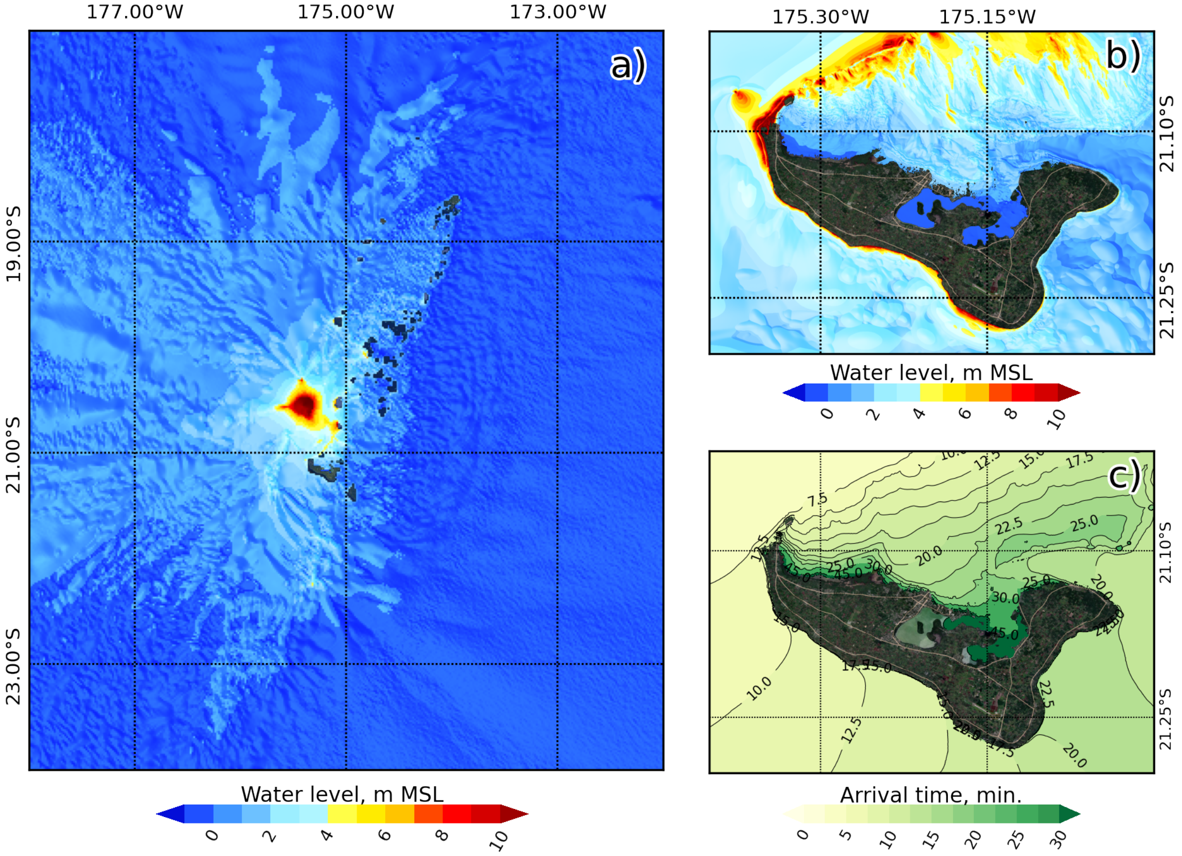

Fig. S5 (Cont.). Tsunami simulation results for the 40.0 Mt with the deep sea depth condition; a) Maximum water level on 1st region, b) Maximum water level on 3rd region, and c) Arrival time on 3rd region. The map was created with a QGIS software, version 3.16.15-Hannover (http://www.qgis.org), and the satellite image for basemap was downloaded from QuickMapServices plugin (https://github.com/nextgis/quickmapservices) through the QGIS.

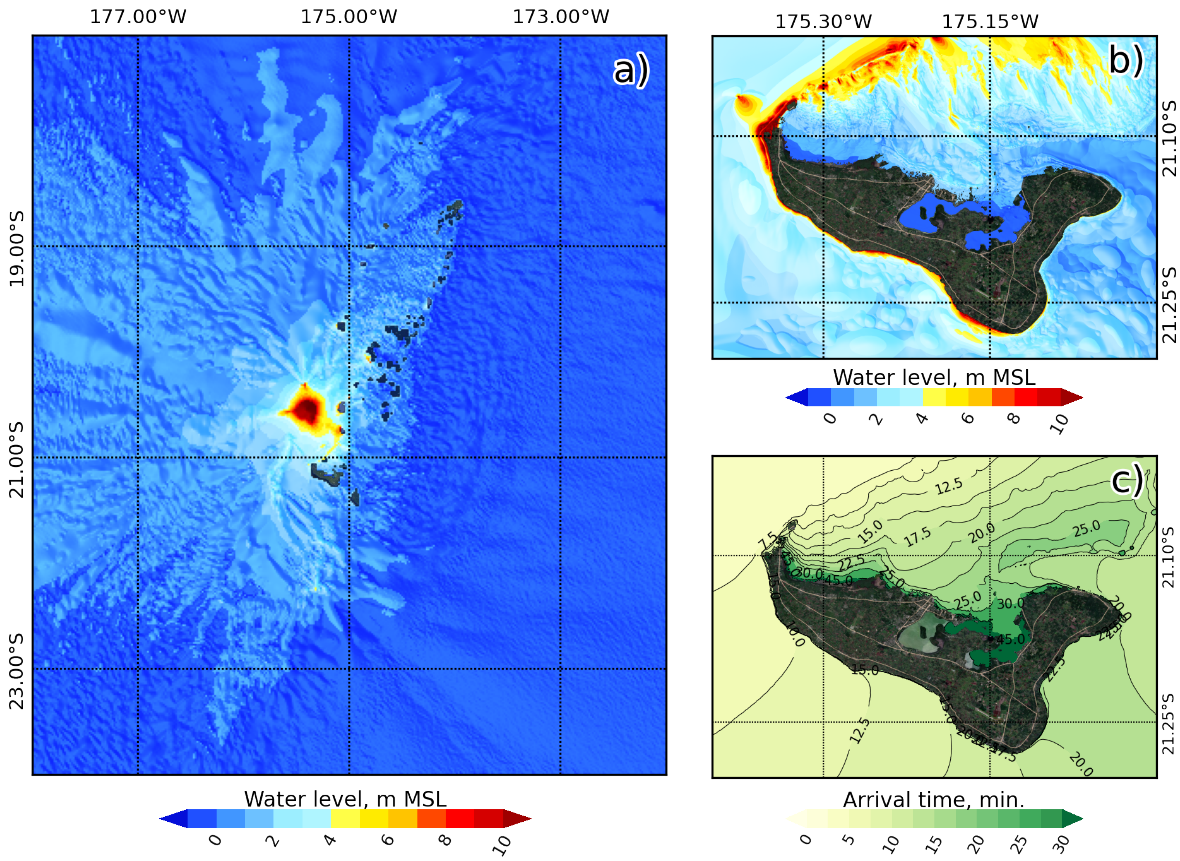

Fig. S5 (Cont.). Tsunami simulation results for the 45.0 Mt with the deep sea depth condition; a) Maximum water level on 1st region, b) Maximum water level on 3rd region, and c) Arrival time on 3rd region. The map was created with a QGIS software, version 3.16.15-Hannover (http://www.qgis.org), and the satellite image for basemap was downloaded from QuickMapServices plugin (https://github.com/nextgis/quickmapservices) through the QGIS.

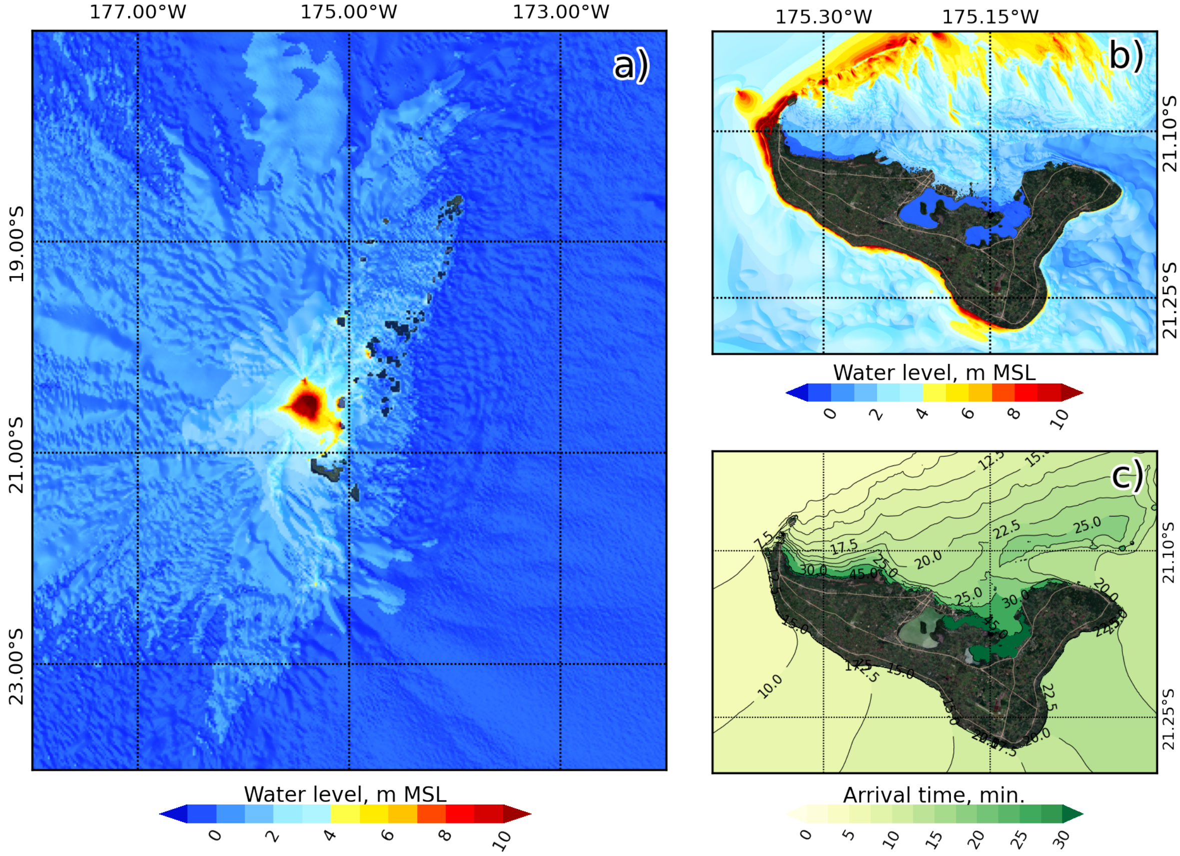

Fig. S5 (Cont.). Tsunami simulation results for the 50.0 Mt with the deep sea depth condition; a) Maximum water level on 1st region, b) Maximum water level on 3rd region, and c) Arrival time on 3rd region. The map was created with a QGIS software, version 3.16.15-Hannover (http://www.qgis.org), and the satellite image for basemap was downloaded from QuickMapServices plugin (https://github.com/nextgis/quickmapservices) through the QGIS.

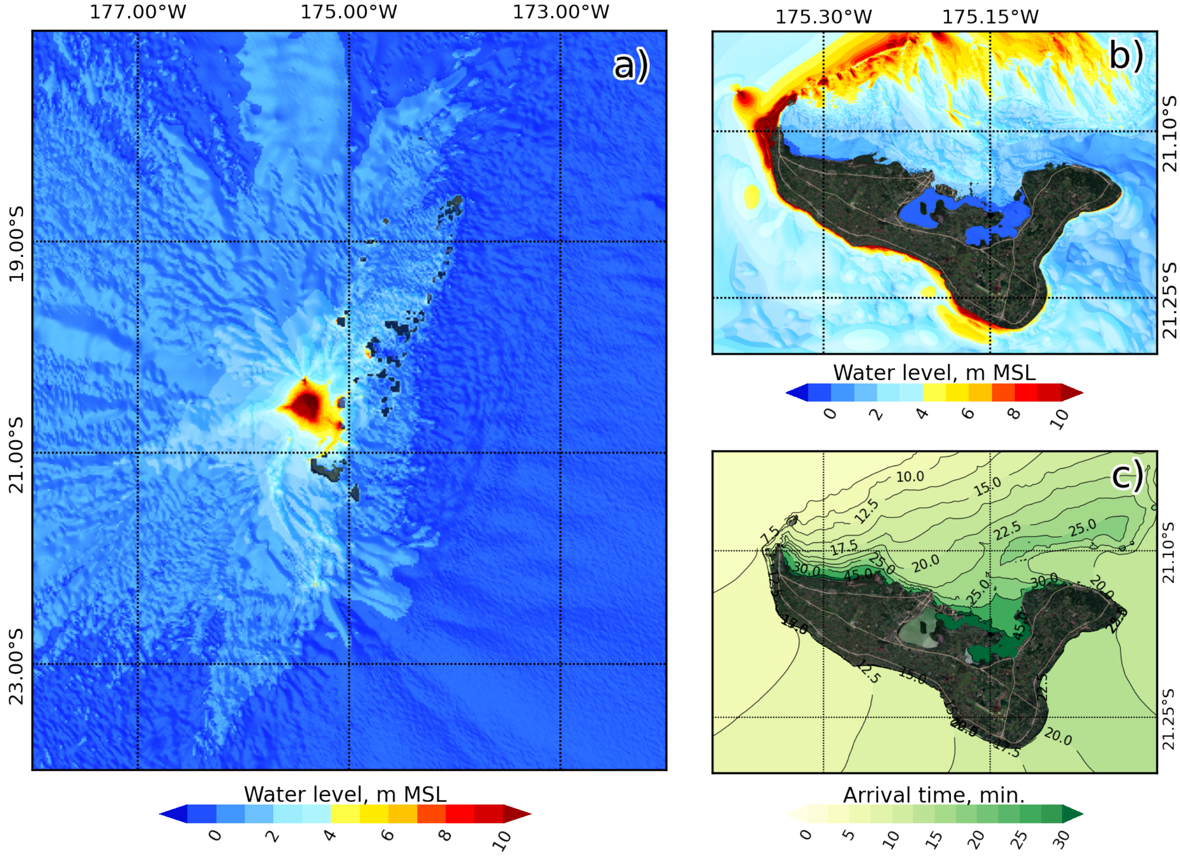

Fig. S5 (Cont.). Tsunami simulation results for the 55.0 Mt with the deep sea depth condition; a) Maximum water level on 1st region, b) Maximum water level on 3rd region, and c) Arrival time on 3rd region. The map was created with a QGIS software, version 3.16.15-Hannover (http://www.qgis.org), and the satellite image for basemap was downloaded from QuickMapServices plugin (https://github.com/nextgis/quickmapservices) through the QGIS.

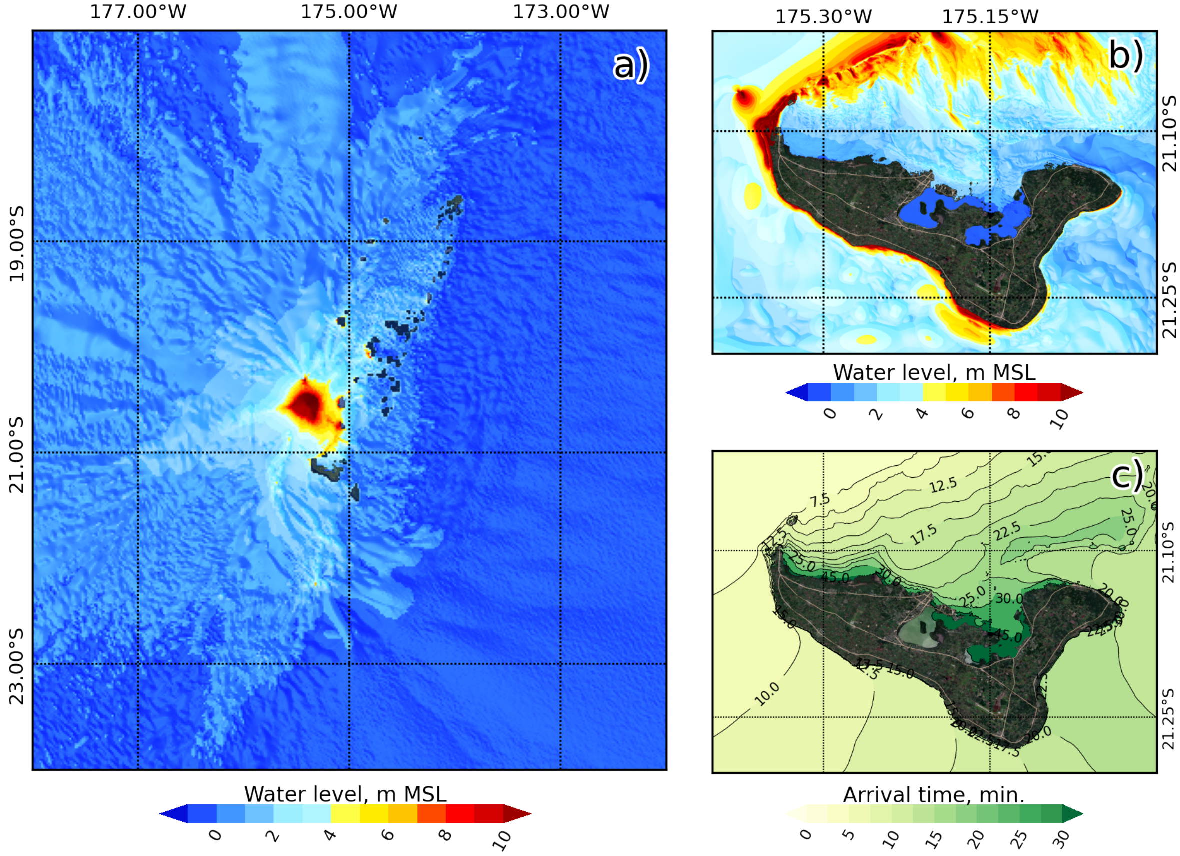

Fig. S5 (Cont.). Tsunami simulation results for the 60.0 Mt with the deep sea depth condition; a) Maximum water level on 1st region, b) Maximum water level on 3rd region, and c) Arrival time on 3rd region. The map was created with a QGIS software, version 3.16.15-Hannover (http://www.qgis.org), and the satellite image for basemap was downloaded from QuickMapServices plugin (https://github.com/nextgis/quickmapservices) through the QGIS.

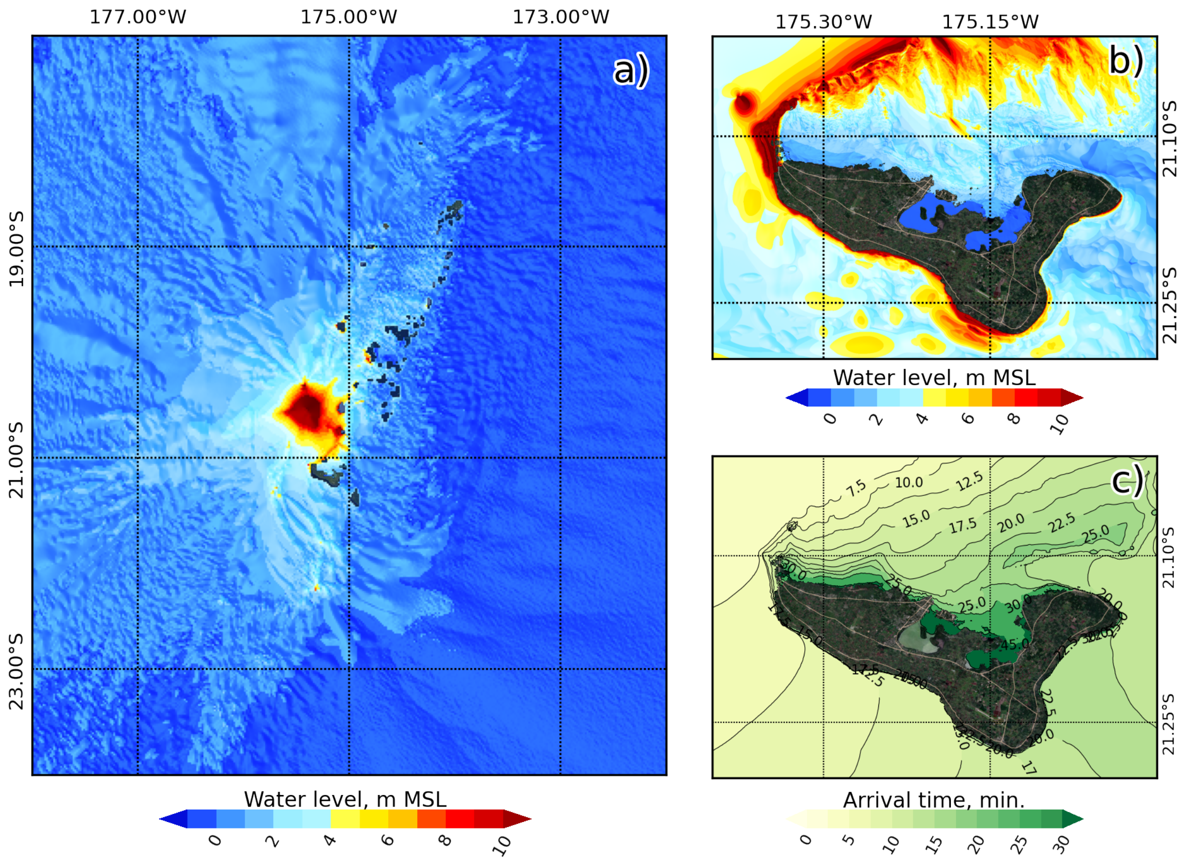

Fig. S5 (Cont.). Tsunami simulation results for the 75.0 Mt with the deep sea depth condition; a) Maximum water level on 1st region, b) Maximum water level on 3rd region, and c) Arrival time on 3rd region. The map was created with a QGIS software, version 3.16.15-Hannover (http://www.qgis.org), and the satellite image for basemap was downloaded from QuickMapServices plugin (https://github.com/nextgis/quickmapservices) through the QGIS.

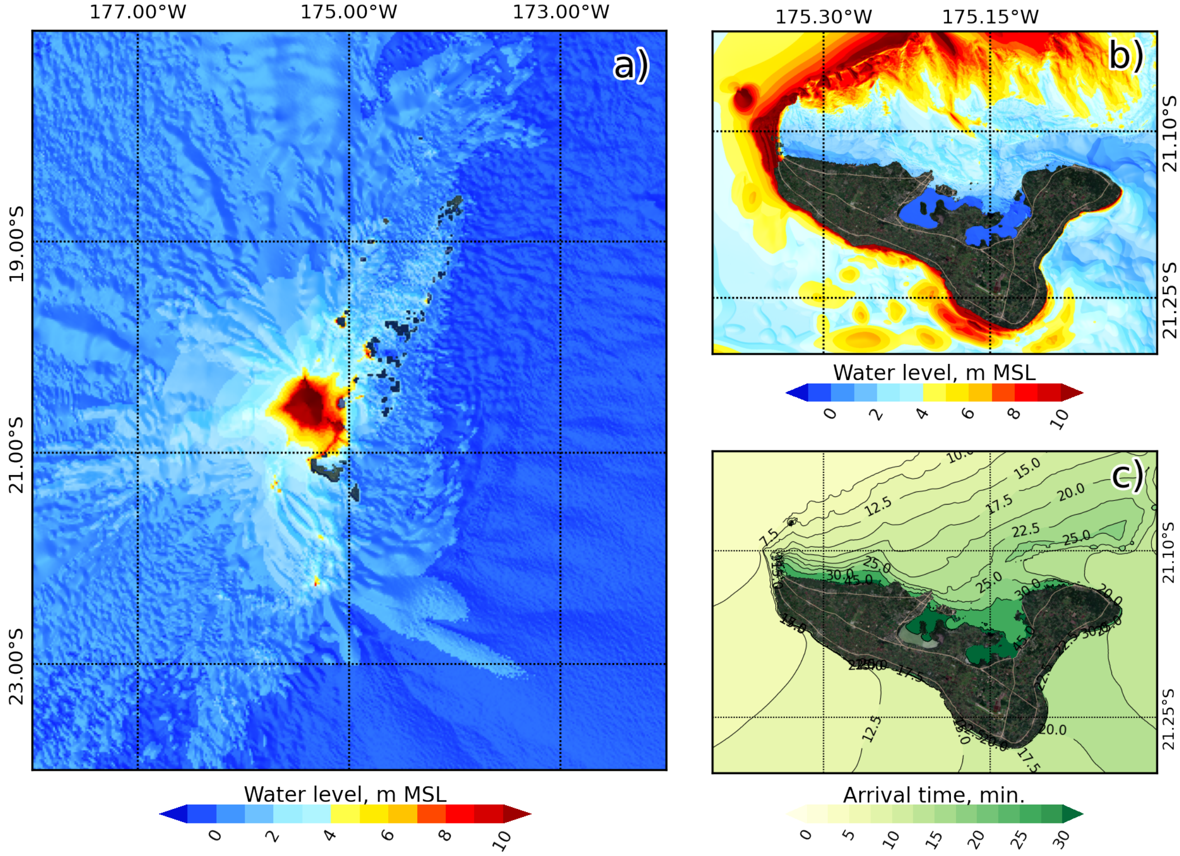

Fig. S5 (Cont.). Tsunami simulation results for the 90.0 Mt with the deep sea depth condition; a) Maximum water level on 1st region, b) Maximum water level on 3rd region, and c) Arrival time on 3rd region. The map was created with a QGIS software, version 3.16.15-Hannover (http://www.qgis.org), and the satellite image for basemap was downloaded from QuickMapServices plugin (https://github.com/nextgis/quickmapservices) through the QGIS.

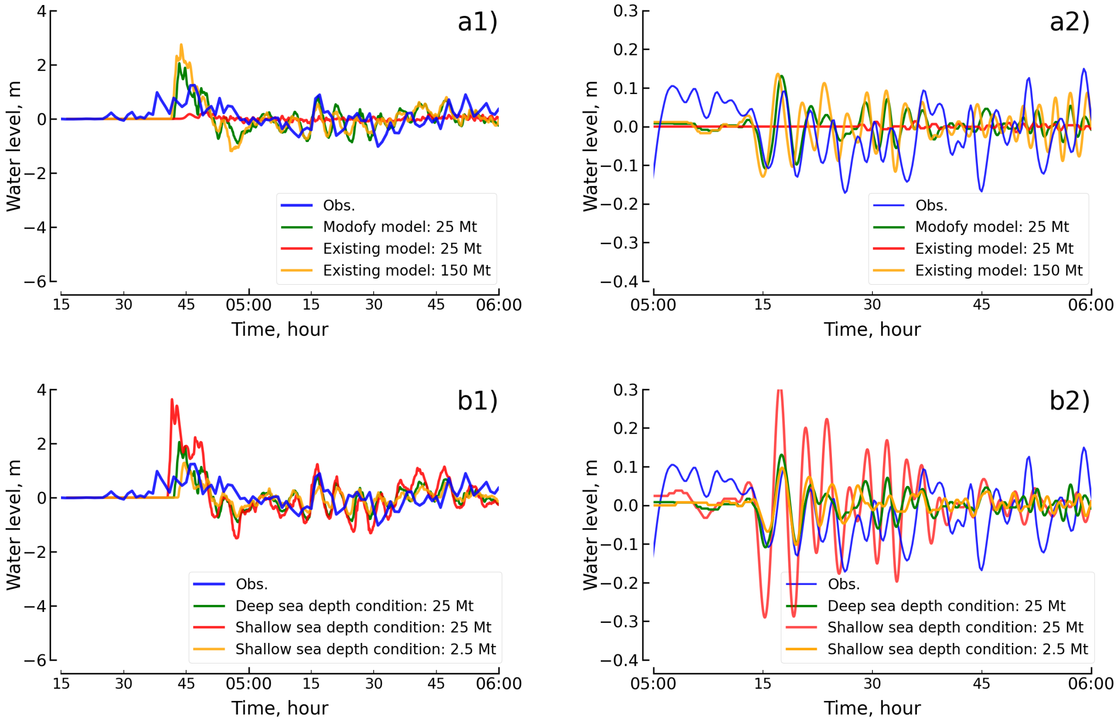

Fig. S6. Sensitivity of the proposed model in reproducing the waveform, showing the Nuku'alofa tide gauge in 1st column and the NZG DART in 2nd column. a) Comparison between the existing and modified empirical models. b) Comparison between the deep sea depth condition and shallow sea depth condition.

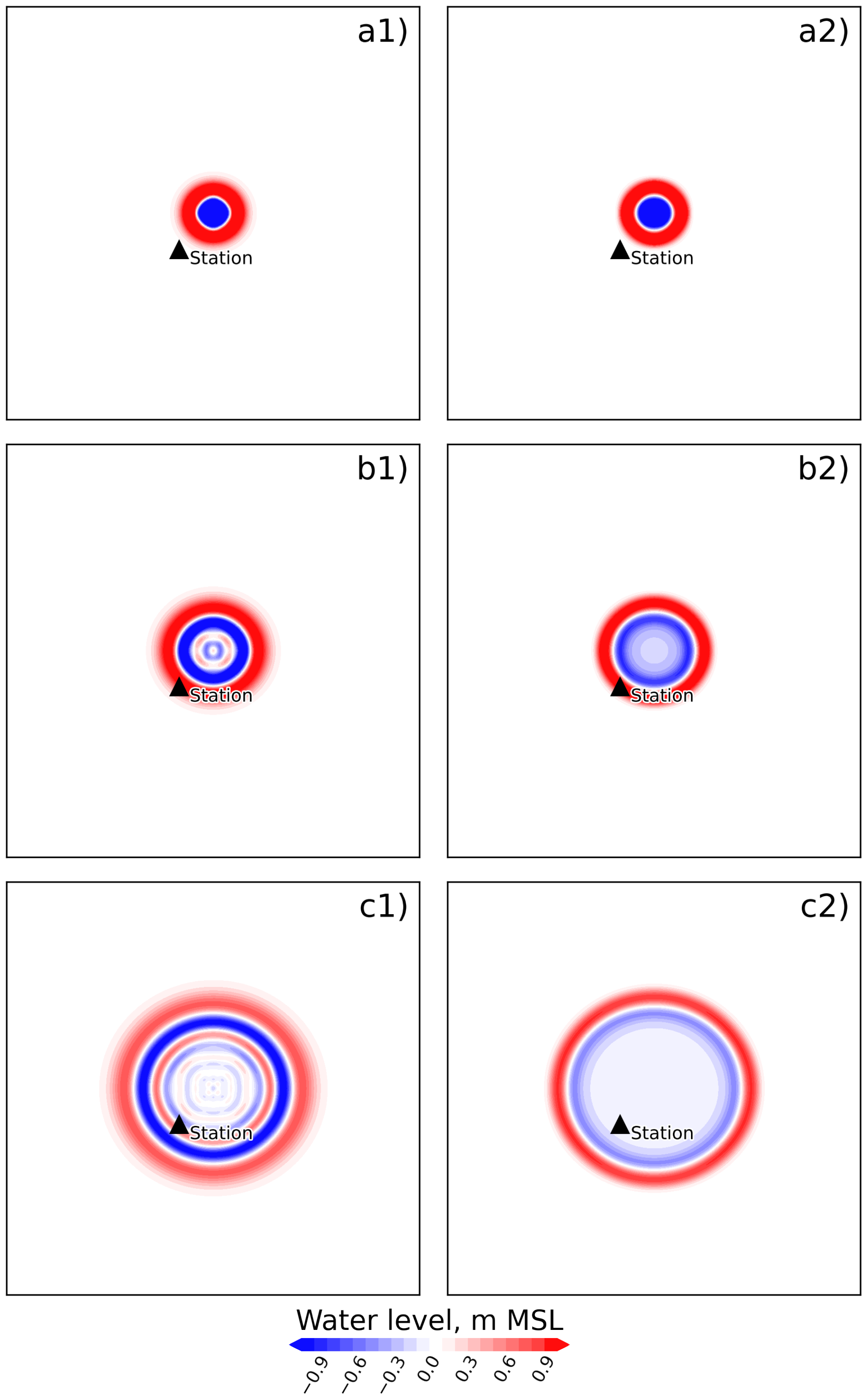

Fig. S7. Comparison of the wave distribution with (1st column) and without (2nd column) the dispersive effect: a) 2 min.; b) 4 min.; c) 8 min; d) 12 min.; e) 15 min.; and f) 17 min.

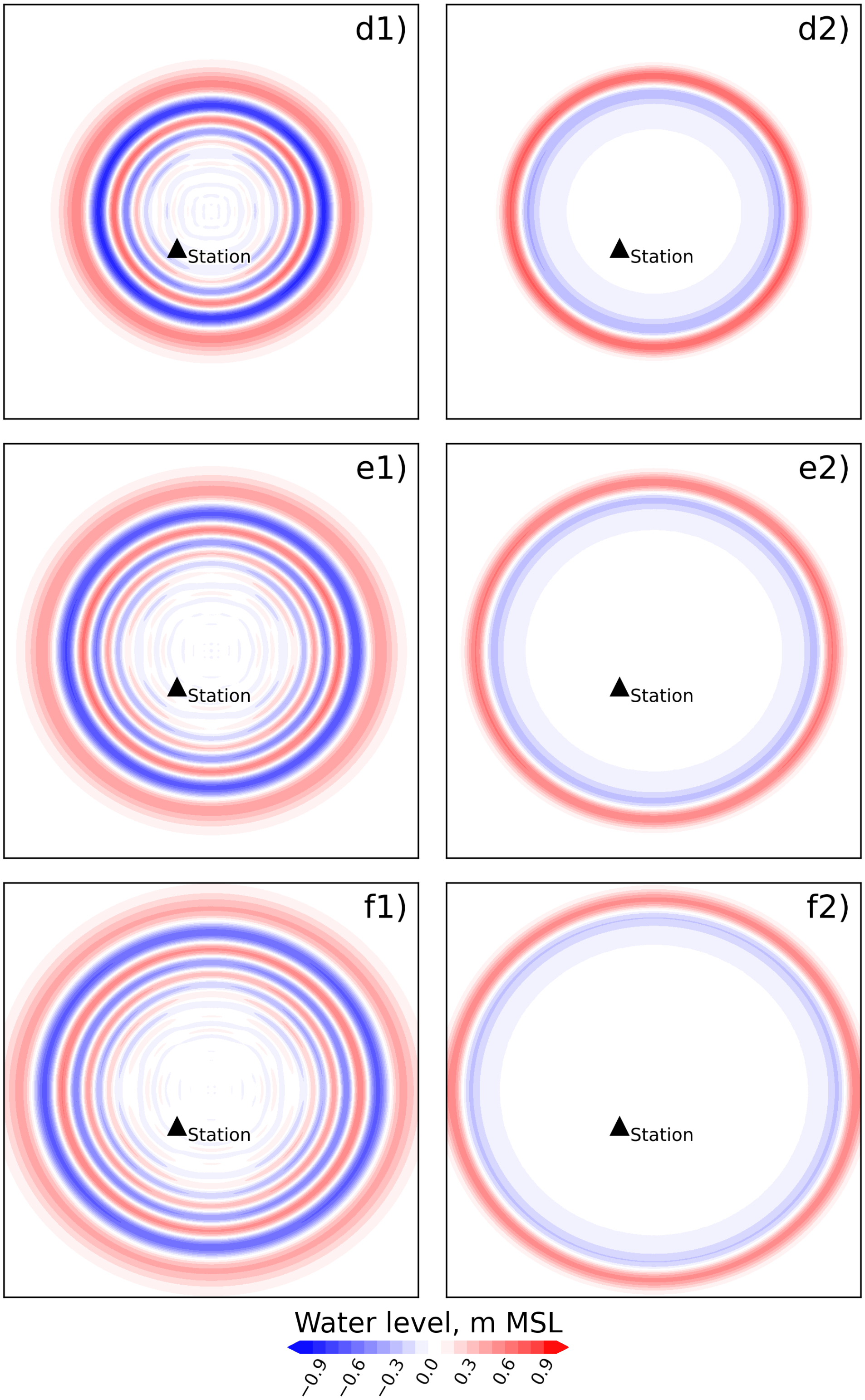

Fig. S7 (Cont.). Comparison of the wave distribution with (1st column) and without (2nd column) the dispersive effect: a) 2 min.; b) 4 min.; c) 8 min; d) 12 min.; e) 15 min.; and f) 17 min.

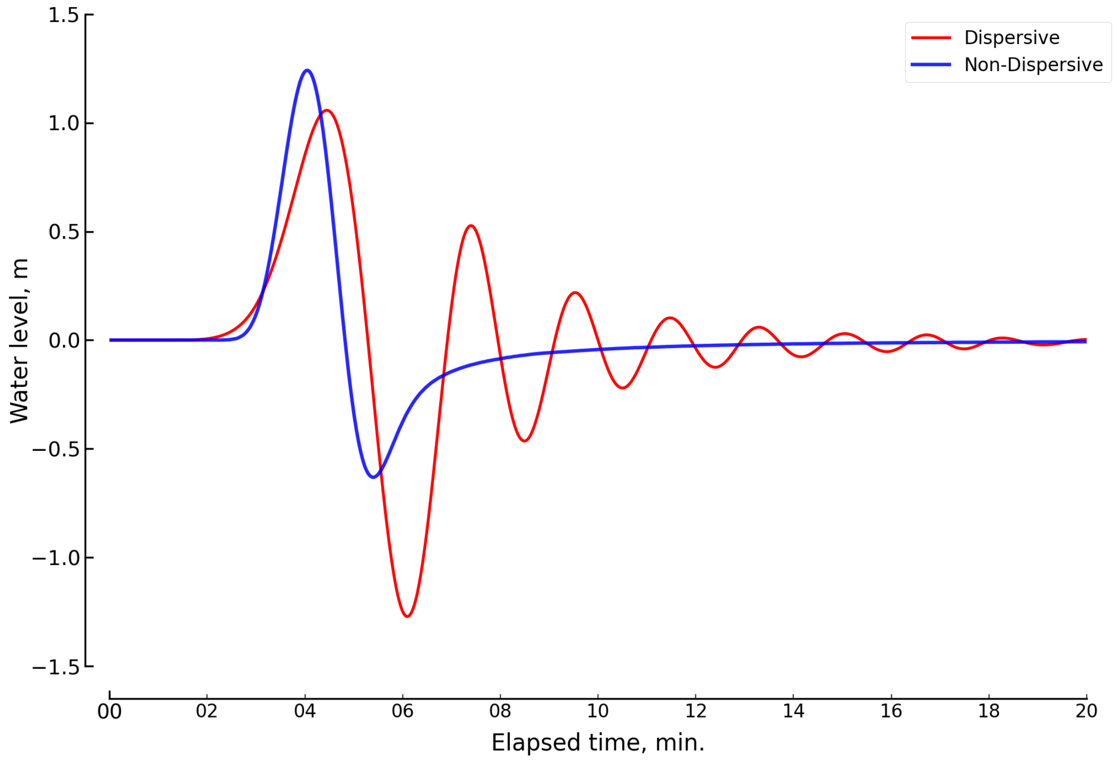

Fig. S8. Comparison of the waveform with and without the dispersive effect on the observed station (Triangle point in Figure S7).

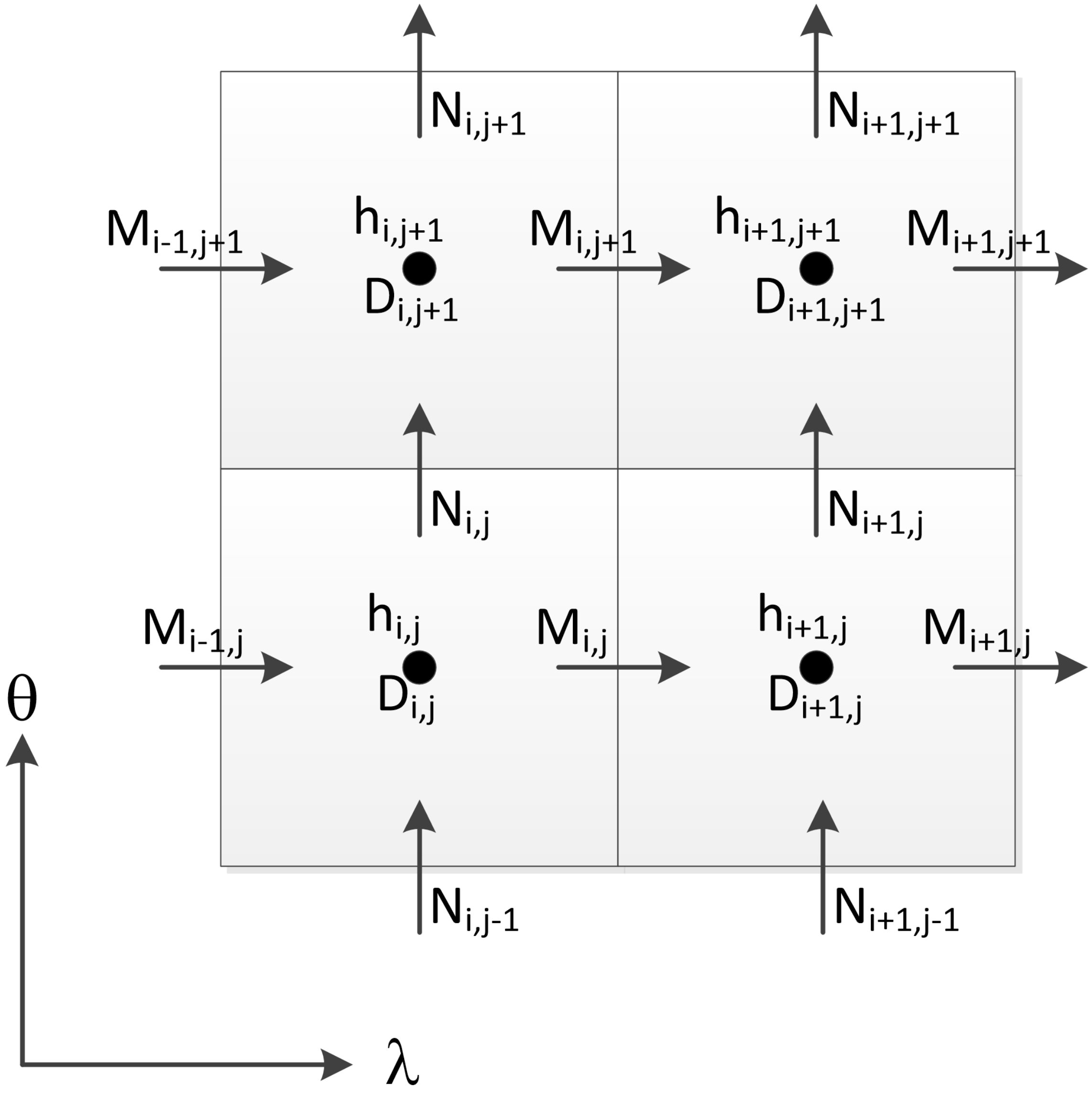

Fig. S9. Staggered-grid system for the finite difference method for tsunami simulation with nonlinear dispersive equations.
